# MicroRNA-133 Inhibits Behavioral Aggregation by Controlling Dopamine Synthesis in Locusts

**DOI:** 10.1371/journal.pgen.1004206

**Published:** 2014-02-27

**Authors:** Meiling Yang, Yuanyuan Wei, Feng Jiang, Yanli Wang, Xiaojiao Guo, Jing He, Le Kang

**Affiliations:** 1 State Key Laboratory of Integrated Management of Pest Insects and Rodents, Institute of Zoology, Chinese Academy of Sciences, Beijing, China; 2 Institute of Applied Biology, Shanxi University, Taiyuan, Shanxi, China; 3 Beijing Institutes of Life Science, Chinese Academy of Sciences, Beijing, China; Janelia Farm Research Campus, Howard Hughes Medical Institute, United States of America

## Abstract

Phenotypic plasticity is ubiquitous and primarily controlled by interactions between environmental and genetic factors. The migratory locust, a worldwide pest, exhibits pronounced phenotypic plasticity, which is a population density-dependent transition that occurs between the gregarious and solitary phases. Genes involved in dopamine synthesis have been shown to regulate the phase transition of locusts. However, the function of microRNAs in this process remains unknown. In this study, we report the participation of miR-133 in dopamine production and the behavioral transition by negatively regulating two critical genes, *henna* and *pale*, in the dopamine pathway. miR-133 participated in the post-transcriptional regulation of *henna* and *pale* by binding to their coding region and 3′ untranslated region, respectively. miR-133 displayed cellular co-localization with *henna*/*pale* in the protocerebrum, and its expression in the protocerebrum was negatively correlated with *henna* and *pale* expression. Moreover, miR-133 agomir delivery suppressed *henna* and *pale* expression, which consequently decreased dopamine production, thus resulting in the behavioral shift of the locusts from the gregarious phase to the solitary phase. Increasing the dopamine content could rescue the solitary phenotype, which was induced by miR-133 agomir delivery. Conversely, miR-133 inhibition increased the expression of *henna* and *pale*, resulting in the gregarious-like behavior of solitary locusts; this gregarious phenotype could be rescued by RNA interference of *henna* and *pale*. This study shows the novel function and modulation pattern of a miRNA in phenotypic plasticity and provides insight into the underlying molecular mechanisms of the phase transition of locusts.

## Author Summary

Phenotypic plasticity refers to the ability of an organism to alter its phenotypes in response to environmental changes. Genetic factors, such as coding and non-coding RNAs, contribute to phenotypic variation. MicroRNAs (miRNAs), which are non-coding RNAs, function as post-transcriptional repressors of gene expression. Migratory locusts show remarkable phenotypic plasticity, referred to as phase transition, which is dependent on population density changes. In the present study, we elucidated the miRNA-133-mediated post-transcriptional mechanisms involved in dopamine production that result in behavioral phase changes. We found that miR-133 directly represses two genes, *henna* and *pale*, in the dopamine pathway. Administration of the miR-133 agomir decreased dopamine production and induced a behavioral shift from the gregarious to the solitary phase. Additionally, miR-133 targeted *henna* in the coding region and *pale* in the 3′ untranslated region, possibly indicating that different mechanisms of post-transcriptional regulation by miR-133 occur in the dopamine pathway. Moreover, the rescue experiments significantly eliminated the effects of miR-133 overexpression and inhibition on the behavioral phase changes of locusts. Our results demonstrate the role of miR-133 in phenotypic plasticity in locusts, in which the miR-133 regulates behavioral changes by controlling dopamine synthesis.

## Introduction

The phenotypes of an organism are directly controlled by the underlying interactions between genetic and environmental factors [1]. Environmental factors significantly contribute to the formation and development of phenotypic plasticity in living organisms [2]. The migratory locust *Locusta migratoria*, a worldwide insect pest, shows remarkable phenotypic plasticity, called phase transition, which is dependent on population density changes [3], [4]. Locust plague outbreaks are triggered by phase transition, during which locusts transform from the solitary to the gregarious phase, thus forming large, fast-flying swarms [4], [5]. Previous studies on the expression and regulation of protein-coding genes have revealed the molecular regulatory mechanisms of phase changes in the migratory locust. A subset of primary phase-determining genes, including *henna*, *pale*, and *vat1* in the dopamine pathway, *CSP* and *takeout* involved in olfactory sensitivity, and carnitine acetyltransferase and palmitoyl transferase from the carnitine system, have been shown to mediate the behavioral phase change in locusts [6]–[9]. However, non-coding RNAs that can mediate the molecular mechanisms of phenotypic plasticity associated with locust phase polyphenism have not been studied.

MicroRNAs (miRNAs), small non-coding regulatory RNAs (∼22 nucleotides), are a class of identified genetic factors that post-transcriptionally regulate gene expression. In plants, miRNAs can trigger the endonucleolytic cleavage of mRNA by targeting perfect or nearly perfect complementary sites located in the 5′ untranslated regions (UTRs), coding regions, or 3′UTRs of the target genes [10], [11]. In animals, the majority of miRNA activity leads to translational repression or mRNA degradation by a low complementary base-pairing with the 3′UTRs of the target genes [12], [13]. However, a few experimental studies have shown miRNA target sites in the coding regions of genes in animals [14]. A single miRNA may target multiple mRNAs, and a single mRNA may have binding sites for multiple miRNAs [15]. This feature creates a complex regulatory system for biological processes, such as cell growth, proliferation, differentiation, development, apoptosis, stress responses, and disease pathogenesis [15]–[18]. Interactions between miRNAs and environmental factors can critically affect phenotypes, thus resulting in phenotypic plasticity. For example, miR-7 in *Drosophila* buffers gene expression against fluctuating temperature conditions during development [19]. Several studies have investigated the responses of vertebrates and insects to environmental stresses such as population density and stress, freeze tolerance, anoxia tolerance, and hibernation [20]–[23]. However, the involvement of miRNAs in animal adaptation or phenotypic plasticity in response to environmental stress has not yet been fully elucidated. Given the increasing importance of studies investigating the relationship between environmental factors and miRNA, the elucidation of the functional roles of individual miRNAs in response to environmental stresses is necessary. Significant differences in miRNA profiles between gregarious and solitary locusts were identified in our previous study [24], in which we indicated that miRNAs are potentially involved in the phase transition. However, the mechanism by which miRNA regulates the phase transition remains unexplored in the migratory locust.

In the present study, we used the migratory locust as a model to study the relationship between environmental factors mediating phenotypic plasticity and miRNA function. Using bioinformatic prediction algorithms and experimental confirmation, we found that the brain-expressed miR-133 directly targets key genes (*henna* and *pale*) in the dopamine biosynthetic pathway. miR-133 regulates dopamine production during locust phase transition by targeting these genes in response to population density stresses. *In vivo* gain- and loss-of-function studies indicate that miR-133 is necessary to maintain the solitary behavioral features of the migratory locust. These data demonstrate that miR-133 is critical in the phase transitions of the migratory locust.

## Results

### miR-133 potentially targets the dopamine pathway

**Figure 1:**
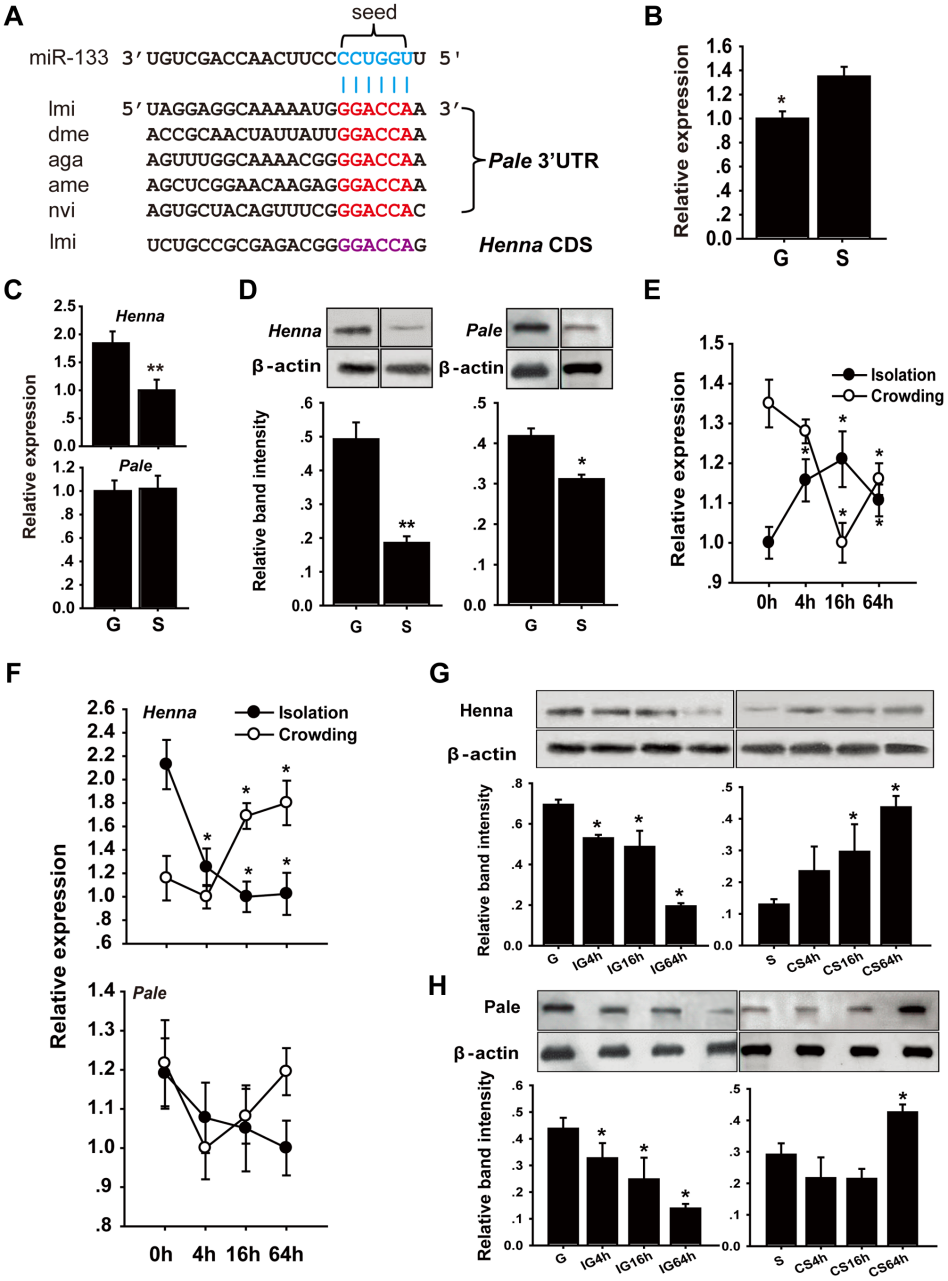
The dopamine pathway may be targeted by miR-133. (A) miR-133 target sites were predicted in the pale and henna genes of Locusta migratoria (lmi), Drosophila species (dme), Anopheles gambiae (aga), Apis mellifera (ame), and Nasonia vitripennis (nvi). (B) The expression levels of miR-133 were determined in the brains of fourth instar gregarious (G) and solitary (S) locust nymphs using qPCR. (C, D) The expression levels of henna and pale were determined in the brains of fourth instar gregarious and solitary locust nymphs using qPCR and western blot analyses. (E) The expression levels of miR-133 were determined in the brains of fourth instar gregarious nymphs after isolation (IG) and in solitary nymphs after crowding (CS) using qPCR. (F–H) The expression levels of henna and pale were determined in the brains of fourth instar nymphs over the course of IG and CS using qPCR and western blot analyses. The qPCR data are presented as the mean ± SEM (n  =  6). The western blot bands were quantified using densitometry and are expressed as the mean ± SEM (n  =  4). *p<0.05; **p<0.01.

Conservation of orthologous miRNA genes is an important criterion for the identification of potential miRNA functional targets [25]. Thus, we hypothesized that the miRNA-dependent regulatory mechanism of the dopamine pathway was conserved between locusts and other insects. Using the TargetScan database, we analyzed *Drosophila* for miRNAs that could potentially bind to the 3′UTR of *pale*, which is a critical gene in the dopamine biosynthesis pathway [6]. Eight miRNAs exhibited highly conserved target sites, and 16 miRNAs possessed poorly conserved target sites located in the 3′UTR of *pale* in *Drosophila* (Table S1 and Figure S1). miR-133 and miR-994 target sites, which belong to conserved and poorly conserved groups, respectively, were also located in the 3′UTR of *pale* in locusts and other insects (Table S2), including *Anopheles gambiae*, *Apis mellifera*, and *Nasonia vitripennis* (Figure 1A). No miR-133 or miR-994 target sites were predicted in the 3′UTR or coding region of *henna*, which is another critical gene involved in the dopamine pathway in *Drosophila*. In contrast, a miR-133 target site was predicted in the coding region of *henna* in locusts (Figure 1A and Figure S1).

We performed stem-loop quantitative reverse transcriptase-polymerase chain reaction (qRT-PCR) to quantify the miR-133 and miR-994 expression levels in the brains of gregarious and solitary locusts. The miR-133 expression level in gregarious locusts was significantly lower than that in solitary locusts (Figure 1B), whereas no significant change was found in the miR-994 expression levels between the gregarious and solitary locusts (Figure S2). We also determined the mRNA and protein expression levels of *henna* and *pale* in the locust brain. The mRNA expression level of *henna* was higher in the gregarious locusts than in the solitary locusts, whereas no difference was found in the mRNA expression of *pale* (Figure 1C). However, both *henna* and *pale* exhibited significantly higher protein expression in the gregarious locusts than in the solitary locusts (Figure 1D). Moreover, isolating the gregarious locusts (IG) resulted in a significant increase in the miR-133 expression levels, with the highest level observed at 16 h; in contrast, crowding the solitary locusts (CS) resulted in a significant decrease in miR-133 expression (Figure 1E). The protein and mRNA expression levels of *henna* were down-regulated by the IG and up-regulated by the CS (Figures 1F and 1G). Similarly, the protein expression of *pale* was suppressed by the IG and promoted by the CS (Figure 1H). However, no obvious change in *pale* mRNA expression was observed at the mRNA level during the IG and the CS, respectively (Figure 1F). These results indicate that miR-133 expression is negatively correlated with *henna* and *pale* protein expression during the phase transition of locusts.

### *Henna* and *pale* are direct targets of miR-133

**Figure 2:**
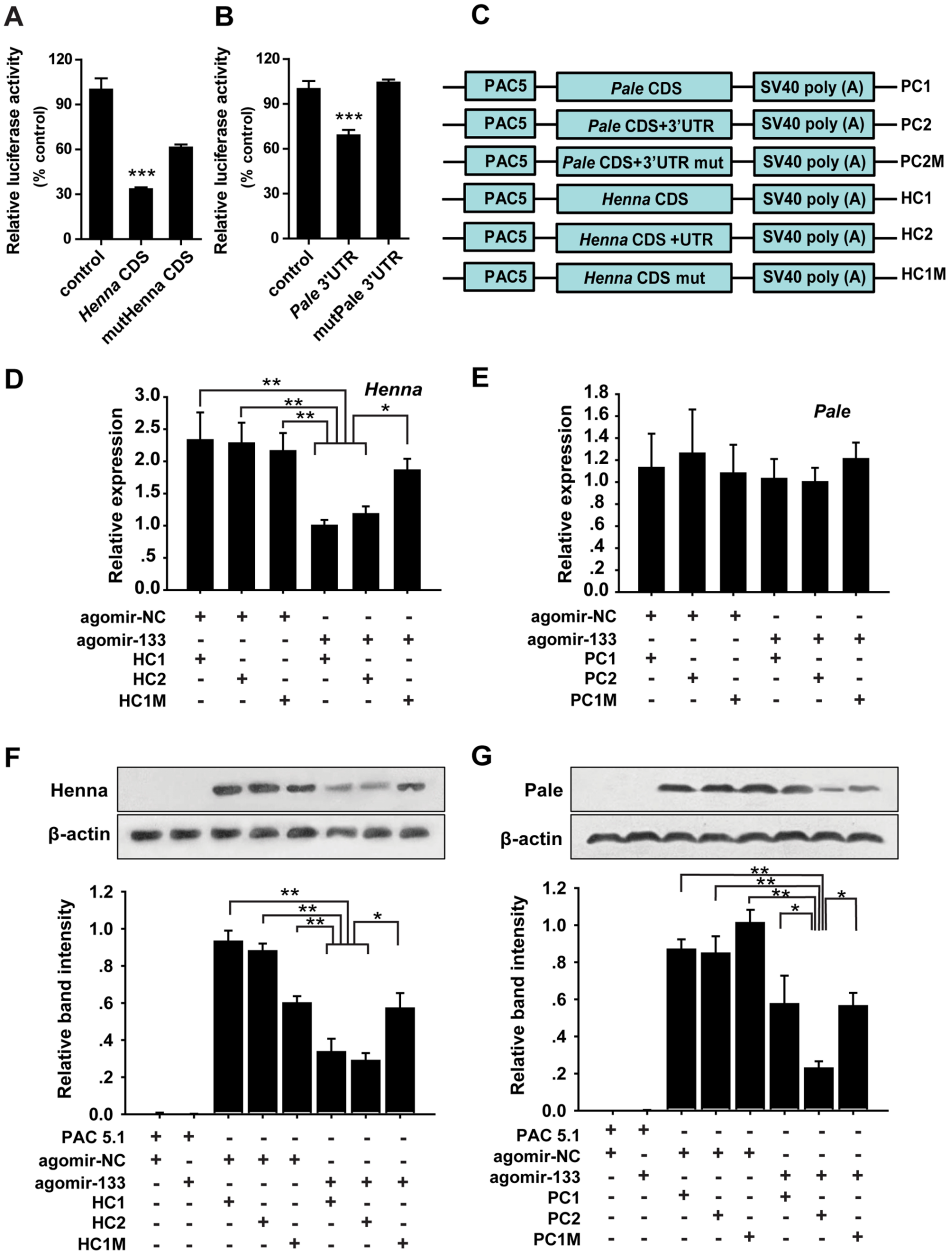
miR-133 targets the coding region of *henna*, but it targets the 3′ UTR of *pale*. (A, B) The interactions between miR-133 and the target binding sites of *henna* (A) and *pale* (B) in migratory locusts were determined using luciferase assays. (C) The strategy used to generate the plasmid expression vectors HC1, HC2, HC1M, PC1, PC2, and PC2M. (D) The mRNA expression levels of *henna* were determined in S2 cells co-transfected with the plasmid expression vectors HC1, HC2, HC1M, and agomir-133 using qPCR. (E) The mRNA expression levels of *pale* were determined in S2 cells co-transfected with the plasmid expression vectors PC1, PC2, PC2M, and agomir-133 using qPCR. (F) The protein expression levels of *henna* were determined in S2 cells co-transfected with the plasmid expression vectors HC1, HC2, HC1M, and agomir-133 by western blot analysis. (G) The protein expression levels of *pale* were determined in S2 cells co-transfected with the plasmid expression vectors PC1, PC2, PC2M, and agomir-133 by western blot analysis. The results were normalized to the expression of *β-actin*. Co-transfection with the empty PAC-5.1/V5 His A vector or the agomir-control (agomir-NC) was used as a negative control. HC1: CDS of *henna*; HC2: both the CDS and 3′UTR of *henna*; HC1M: HC1 containing four seed element mutations in the coding region of *henna*. PC1: CDS of *pale*; PC2: both the CDS and 3′ UTR of *pale*; PC2M: PC2 containing four seed element mutations in the 3′ UTR of *pale*. The data for the luciferase activities and qPCR analyses are presented as the mean ± SEM (*n* = 6). The western blot bands were quantified using densitometry and are expressed as the mean ± SEM (*n* = 4). *p<0.05; **p<0.01.

To confirm the interaction between miR-133 and *henna/pale in vitro*, we performed reporter assays using luciferase constructs fused to the coding region of *henna* and the 3′UTR of *pale*. Compared to the reporter constructs without miR-133 binding sites (controls), the constructs with either the *henna* or *pale* binding sites produced lower luciferase activity when co-transfected with the miR-133 overexpression vectors in S2 cells (Figures 2A and 2B). When the regions homologous to the “seed” sequence of miR-133 were mutated in the *pale* 3′UTR reporter constructs, the luciferase activity returned to the original levels produced by mock transfection with the empty reporter plasmid (Figure 2B). Mutations in the *henna* binding sites resulted in a twofold increase in luciferase activity, although this activity was still lower than that produced by the empty reporter plasmid (Figure 2A). Thus, the predicted target sites in *henna* and *pale* can be targeted by miR-133 in S2 cells.

miRNAs can affect mRNA stability and translation. Therefore, to determine the mechanism of interaction between miR-133 and *henna/pale in vitro*, we studied the mRNA and protein expression levels of *henna* and *pale* using three *henna* and three *pale* expression constructs based on the binding pattern of miR-133 (Figure 2C). In the presence of miR-133 sense oligonucleotides (agomir-133), the introduction of either the coding sequence (CDS) of *henna* alone (HC1) or the CDS and 3′UTR of *henna* (HC2) dramatically suppressed its mRNA and protein expression levels compared to the agomir-control (agomir-NC, Figures 2D and 2F). These suppressions were recovered by mutating the miR-133 binding site in the coding region (HC1M, Figures 2D and 2F), which indicates that the miR-133 binding sites are located in the coding region of *henna*. In contrast, in the presence of agomir-133, the introduction of the 3′UTR of *pale* (PC2) reduced its protein expression but not its mRNA expression. The reduced protein expression was rescued by the mutation of the miR-133 binding sites in its 3′UTR (PC2M, Figures 2E and 2G). However, the coding region alone (PC1) did not affect *pale* expression when co-transfected with agomir-133, compared to agomir-NC (Figures 2E and 2G). Thus, miR-133 participates in the post-transcriptional regulation of *henna* and *pale* by binding to the corresponding coding region and 3′UTR, respectively.

### miR-133 and *henna*/*pale* interact in the locust protocerebrum

**Figure 3:**
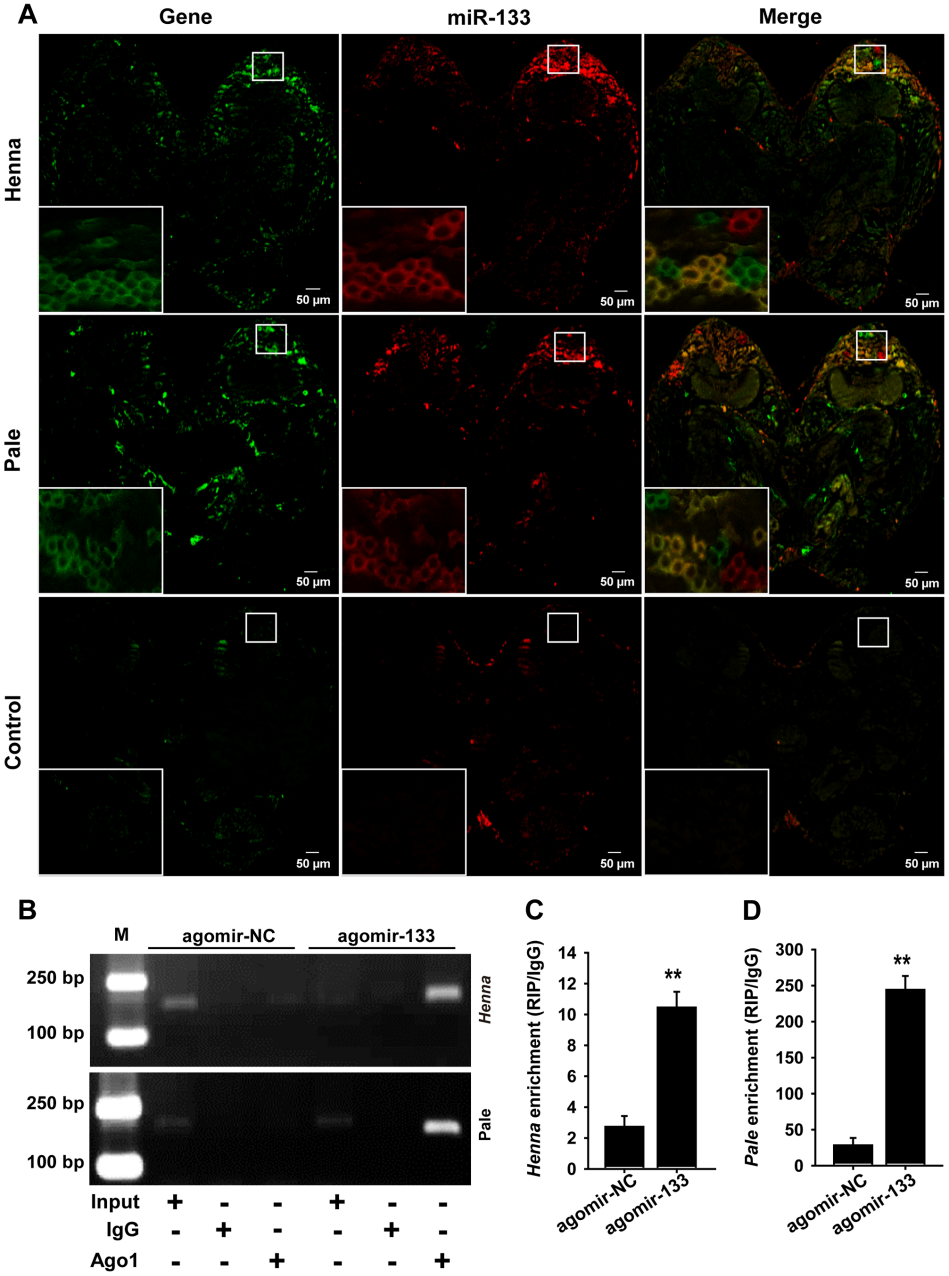
miR-133 can interact with *henna*/*pale* in the locust protocerebrum. (A) The combined *in situ* analyses of miRNA-133 and *henna*/*pale* by the co-labeling of miRNA FISH and immunohistochemistry for miRNA target were conducted to determine the co-localization between these molecules in the locust brain. The squares specifically indicate the areas where miR-133, *henna*, and *pale* were localized in the locust protocerebrum. Where green (*henna* and *pale*) and red signals (miR-133) overlap, a yellow signal is seen, indicating the co-localization of miR-133 and its targets. The images were visualized using an LSM 710 confocal fluorescence microscope (Zeiss) at a magnification of 10× (the small squares) and 63× (the large squares), respectively. (B–D) RIP was performed with an anti-Ago-1 antibody; normal mouse IgG was used as a negative control. RT-PCR (B) or qPCR (C, D) analysis was performed to amplify the *henna* and *pale* mRNA from the Ago-1 immunoprecipitates from extracts of protocerebrum tissue treated with the miR-133 agomir (agomir-133) compared to the agomir-controls (agomir-NC). M, DNA marker. The data for the RIP assay are presented as the mean ± SEM (*n* = 6). **p<0.01.

To determine whether miR-133 co-localized with *henna*/*pale* in the locust brain, we performed combined *in situ* analyses of miRNA-133 and *henn*a/*pale* by the co-labeling of miRNA fluorescence *in situ* hybridization (FISH) and immunohistochemistry miRNA target. We found that miR-133 and *henna* were only detected in the neuronal cell body of the locust protocerebrum (Figures 3A and Figure S3), which is an important region in determining the behavioral responses by output neurons from the mushroom body [26]. *Pale* was also widely expressed in the protocerebrum, including the mushroom body and the neuronal cell body. The results showed that miR-133 is cellular co-localized with *henna*/*pale* in the protocerebrum. Thus, miR-133 and *henna*/*pale* co-localization in the protocerebrum supports the direct interactions between them in the locust brain.

We then performed an RNA immunoprecipitation assay using a monoclonal antibody against the Ago1 protein in the protocerebrum to examine the interaction of miR-133 with *henna*/*pale* (Figures 3B–3D). The results showed that *henna* and *pale* were significantly enriched in the Ago1-immunoprecipitated RNAs from the protocerebrum treated with agomir-133 compared with those from the protocerebrum treated with agomir-NC (Figures 3B–3D). Moreover, we found a significant decrease in miR-133 expression and up-regulation in *pale* and *henna* expression in the protocerebrum of gregarious locusts compared to the levels observed in solitary locusts (Figure S4). These results suggest that the direct regulation of *pale* and *henna* by miR-133 potentially occurs in the locust protocerebrum.

### miR-133 controls dopamine production by targeting *henna* and *pale*

**Figure 4:**
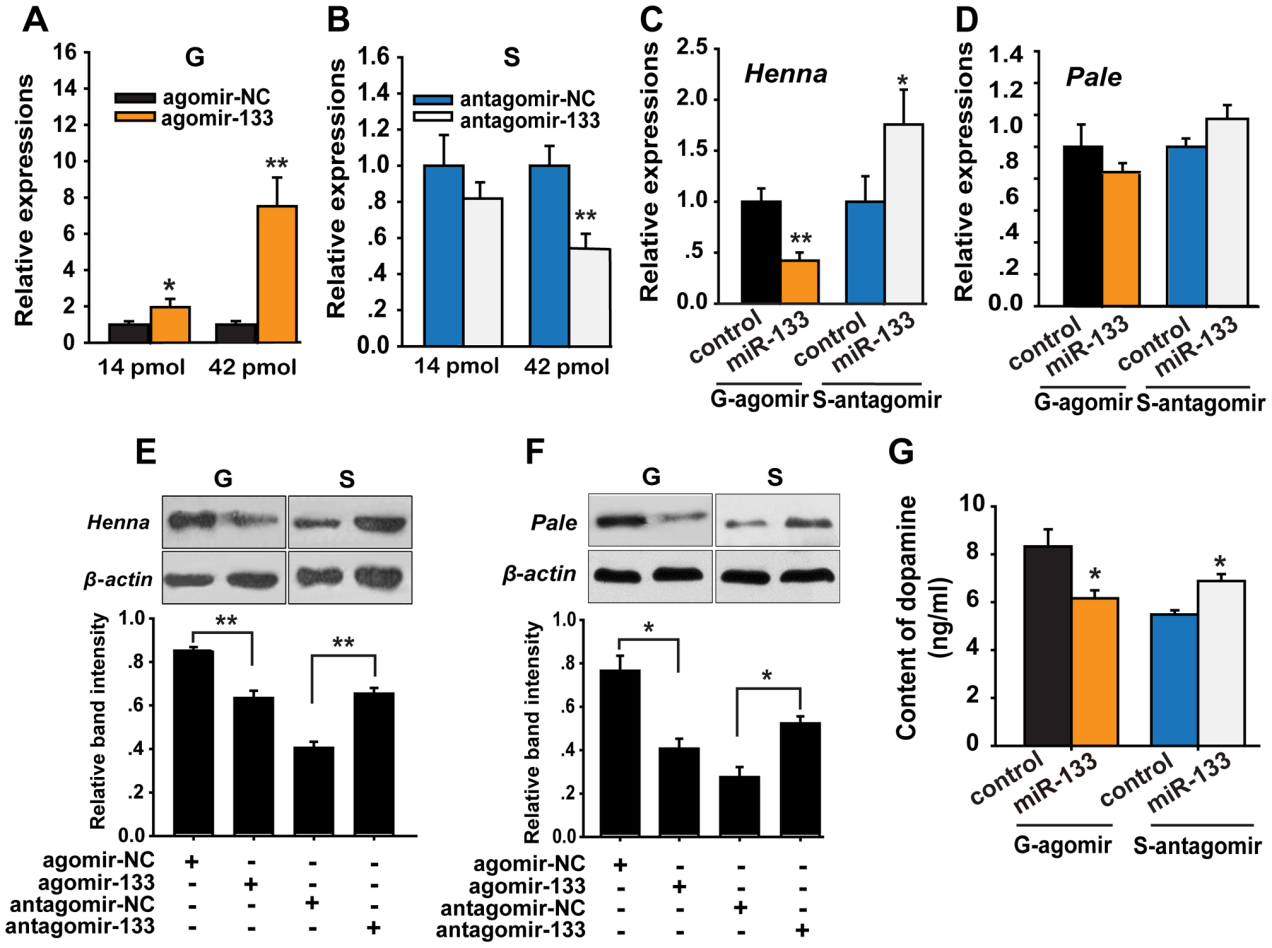
miR-133 controls dopamine production by regulating *henna* and *pale* expression in the locust brain. (A, B) The expression levels of miR-133 were determined 48 h after injection in gregarious locusts (G) and in solitary locusts (S) after treatment with agomir or antagomir, respectively (14 or 42 pmol) using qPCR. (C, D) The effects of 42 pmol of agomir- and antagomir-133 treatment 48 h after injection on the mRNA expression levels of *henna* and *pale* in gregarious and solitary locust brains were studied using qPCR. (E, F) The effects of 42 pmol of agomir- and antagomir-133 48 h after injection on the protein expression levels of *henna* and *pale* in gregarious and solitary locust brains were studied using western blot analysis. (G) Dopamine production after treatment with 42 pmol agomir or antagomir-133 treatment in gregarious and solitary locusts brains was evaluated using HPLC-MS. The qPCR and HPLC-MS data are shown as the mean ± SEM (*n* = 6). The western blot bands were quantified using densitometry and are expressed as the mean ± SEM (*n* = 4). *p<0.05; **p<0.01.

Given that *henna* and *pale* are essential genes in dopamine synthesis, we determined the effect of miR-133 on the dopamine pathway using miR-133 overexpression and knockdown experiments *in vivo*. No obvious change in the miR-133 expression was observed during the fourth instar stage (Figure S5). Thus, nymphs on the second day of the fourth instar stage were randomly assigned to treatment groups, and brain microinjection was performed. We injected agomir-133 or anti-sense oligonucleotides (antagomir-133) to determine the effects of the miR-133 agomir and antagomir treatments. Forty-eight hours after the local microinjection of 14 or 42 pmol of agomir-133, the miR-133 levels dramatically increased in a dose-dependent manner in the gregarious locusts (Figure 4A). Accordingly, antagomir-133 injection elicited the opposite effects on miR-133 levels in solitary locusts, even though 14 pmol of antagomir-133 did not effectively reduce miR-133 expression (Figure 4B). Significant induction or inhibition of miR-133 was also observed 24 h after injection with agomir-133 or antagomir-133, respectively (Figure S6). No significant effects were found on the expression of other miRNAs (Figure S7), including miR-7 and miR-252, which are two miRNAs with high expression in the locust brain (unpublished data), suggesting that the agomir/antagomir injection specifically acted on miR-133. These results reflect the efficiency and specificity of miR-133 manipulation.

To determine the effects of miR-133 on the target genes, we detected the mRNA and protein levels of *henna* and *pale* after agomir-133 or antagomir-133 administration in the locust brain. At 48 h after agomir-133 injection, the mRNA levels of *henna* decreased by approximately 60% in the gregarious locusts when treated with both 42 and 14 pmol compared to those in the control locusts (Figures 4C and S8). Inhibition of *henna* expression was also observed 24 h after agomir-133 injection (Figure S8). At 48 h, injection of 42 pmol of antagomir-133 resulted in miR-133 knockdown, which increased the mRNA expression level of *henna* in the solitary locusts; however, this phenomenon was not observed after injection of 14 pmol antagomir-133 (Figures 4C and S8). In contrast, the mRNA level of *pale* in the locust brain was unaffected by either agomir-133 or antagomir-133 (Figure 4D). Moreover, western blot analysis showed that the protein expression levels of *henna* and *pale* were inhibited by miR-133 agomir administration in the gregarious locusts. Their corresponding protein levels in solitary locusts were up-regulated by miR-133 knockdown (Figures 4E and 4F).

To confirm the effects of *henna* and *pale* expression on dopamine synthesis, we measured dopamine production in the brains of gregarious and solitary locusts using reverse-phase high-performance liquid chromatography (HPLC) with electrochemical detection (ECD) after miR-133 administration. Administration of the miR-133 agomir significantly reduced (approximately 30%, *p*<0.05) dopamine production in the gregarious locusts (Figure 4G). In contrast, miR-133 knockdown significantly increased the dopamine content (approximately 25%, *p*<0.05) of the solitary locusts. These results demonstrate that miR-133 controls dopamine production by regulating the expression of *henna* and *pale* in the locusts.

### miR-133 regulates the phase transition of locusts by fostering solitary behavior

**Figure 5:**
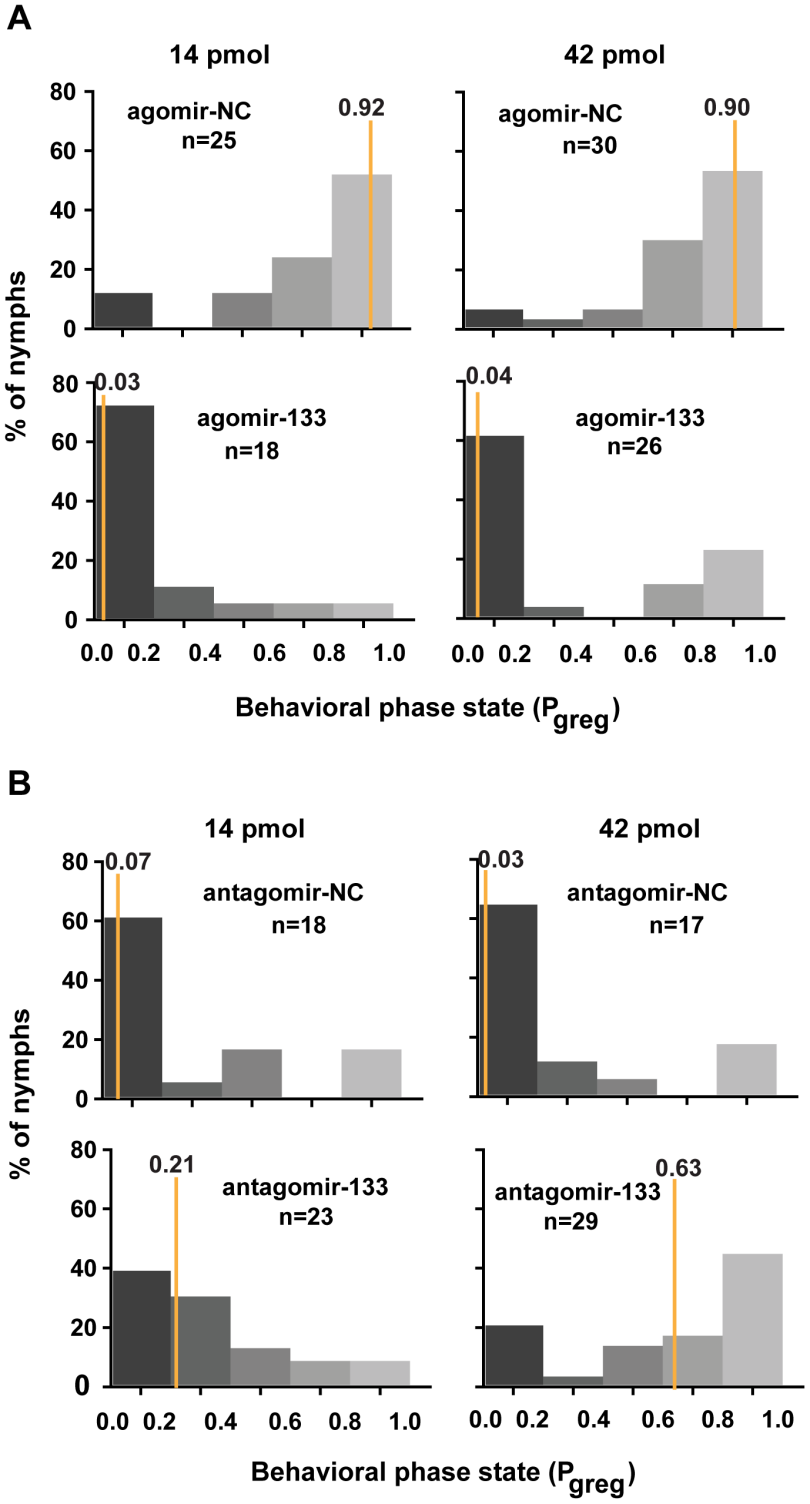
miR-133 fosters the phase transition phenotype of the migratory locust. (A) The effects of 14 or 42 pmol agomir-133 treatment on the behavior of gregarious locusts were studied 48 h after injection. (B) The effects of 14 or 42 pmol antagomir-133 treatment on the behavior of solitary locusts were studied 48 h after injection. P_greg_, probabilistic metric of gregariousness. The vertical lines indicate the median *P*_greg_ values. *P*_greg_ = 1 indicates fully gregarious behavior, and *P*_greg_ = 0 indicates fully solitary behavior.

We reasoned that miR-133 controlled dopamine production by targeting *henna* and *pale* and might modulate the behavioral phase change of locusts. To determine the function of miR-133 during the phase transition, we monitored the behavioral phase changes of gregarious and solitary locusts after agomir-133 and antagomir-133 injection, respectively. The behavior of the gregarious locusts injected with agomir-133 (42 pmol) shifted to the solitary state with a P_greg_ (probabilistic metric of gregariousness) interval of 0 to 0.2; 55% of the gregarious locusts became solitary after 24 h, and 62% became solitary after 48 h, as compared to 15% and 6.7% of the locusts in the agomir-NC group at 24 and 48 h, respectively (Figures 5A and S9). Moreover, the increased miR-133 expression caused by injection with 14 pmol of agomir-133 resulted in a solitary shift at 48-h intervals, with 72% of the injected locusts falling into the P_greg_ interval of 0 to 0.2 (Figure 5A). In parallel, solitary locusts injected with antagomir-133 (42 pmol) exhibited a significant but incomplete shift to gregarious behavior, with 44.8% shifting into the P_greg_ interval of 0.8 to 1.0 (Figure 5B). In contrast, no obvious behavioral change was observed in most of the solitary locusts injected with antagomir-133 (14 pmol) at 48 h intervals (8.7% shifting into the P_greg_ interval of 0.8 to 1.0; Figure 5B). The silencing effects observed after injection of 42 pmol, but not 14 pmol, indicate that the induction of gregarious-like behavior in solitary locusts is dose-dependent, suggesting the feasibility of dose-dependent inhibition of miRNA for effective behavior modulation. These results demonstrate that miR-133 controls the phase transitions in locusts by fostering solitary behavior.

### The dopamine pathway is the direct effector for mediating miR-133-regulated behavioral transition

**Figure 6:**
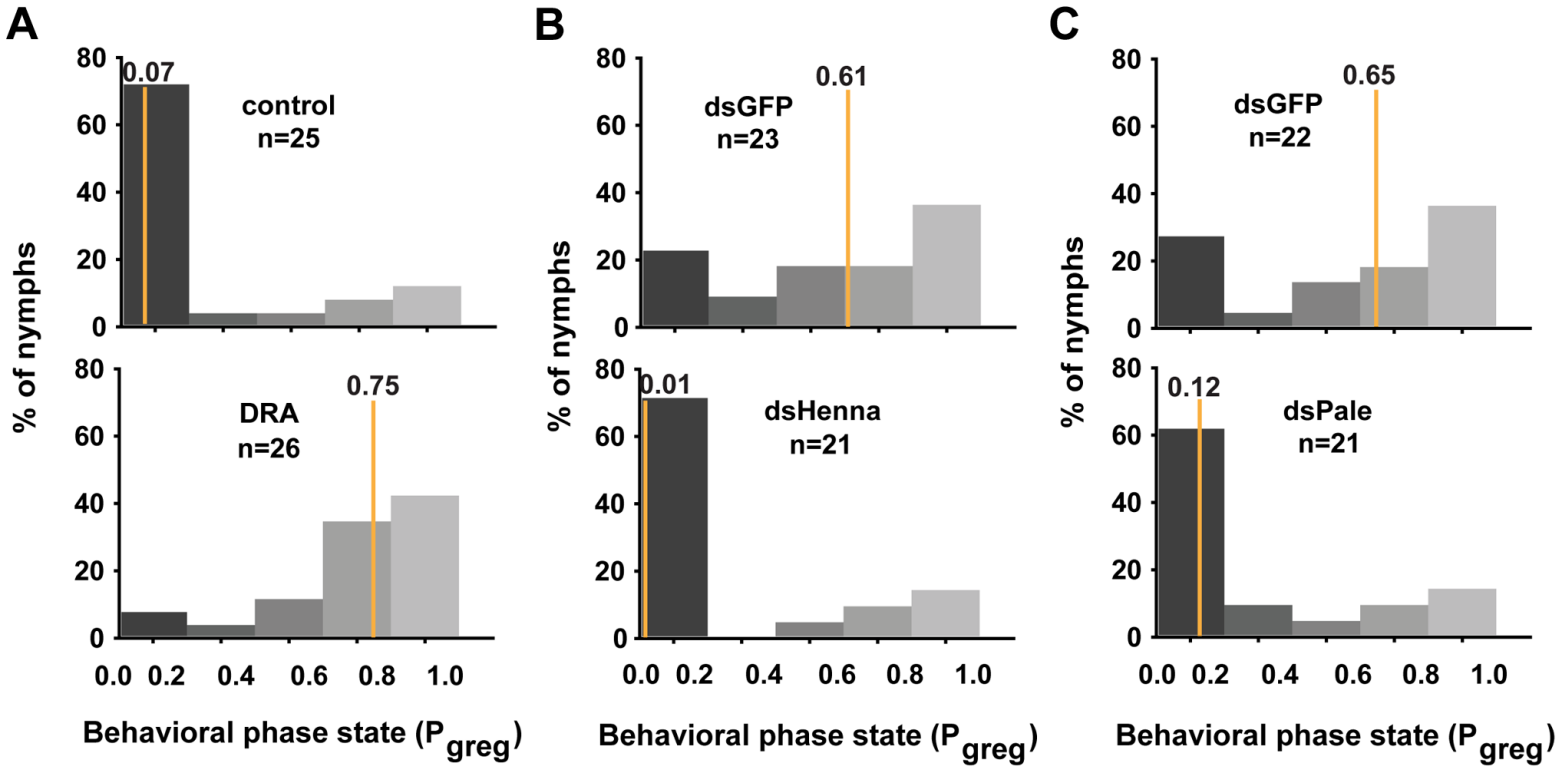
The dopamine pathway is the direct effector for mediating the miR-133-regulated behavioral transition. (A) The behavioral rescue experiment in gregarious locusts was performed by increasing the dopamine content through injection with the dopamine receptor agonist (DRA), *R*-(-)-apomorphine, or a saline control in locusts pre-treated with agomir-133. (B, C) The behavioral rescue experiment in solitary locusts was performed by injecting dsRNA against *henna*/*pale* into the locusts pre-treated with antagomir-133. P_greg_, probabilistic metric of gregariousness. The vertical lines indicate the median P_greg_ values. P_greg_ = 1 indicates fully gregarious behavior, and P_greg_ = 0 indicates fully solitary behavior.

In gregarious locusts, treatment with agomir-133 decreased the dopamine content, thus promoting a significant shift toward the solitary phase. To determine whether the miR-133 delivery-induced decrease in dopamine content was responsible for the behavioral transition, we performed rescue experiments by increasing the dopamine content by injection with *R*-(-)-apomorphine in locusts that were treated with agomir-133. *R*-(-)-apomorphine is a dopamine receptor agonist that can significantly promote gregarious behavioral traits through the dopamine-dependent pathway [27]. The results showed that *R*-(-)-apomorphine injection robustly restored the gregarious behavior in locusts after agomir-133 pre-treatment (P_greg_ = 0.75), compared to the saline-injected controls after 24 h (P_greg_ = 0.07; Figure 6A). Therefore, the change in dopamine content regulated by miR-133 is a key mediator of the behavioral transition between the gregarious and solitary phases.

Treatment of solitary locusts with antagomir-133 resulted in the up-regulation of *henna*/*pale* expression, thereby stimulating gregarious behavioral traits. To determine whether the *henna*/*pale* up-regulation induced by miR-133 knockdown was responsible for the behavioral transition of the locusts, we rescued the behavior phenotypes by injecting double-stranded RNAs (dsRNAs) against *henna*/*pale* into the locusts subjected to antagomir-133 pre-treatment. As expected, the miRNA-133 knockdown-induced behavior phenotype was fully rescued by treatment with dsHenna (P_greg_ = 0.01) or dsPale (P_greg_ = 0.12), compared to the dsGFP-injected controls after 24 h (P_greg_ = 0.61 and P_greg_ = 0.65, respectively; Figures 6B and 6C). Thus, *henna* and *pale* are the key target genes involved the post-transcriptional regulation of miR-133 during the phase transition in locusts.

## Discussion

Our previous studies showed that the *pale*, *henna*, and *vat1* genes, which are involved dopamine synthesis and release, are related to the behavioral phase transitions of the migratory locust [6]. However, the genetic factors that regulate locust phase transition by controlling the expression of these genes remain unknown. In this study, we show that miR-133 controls dopamine production by targeting *henna* and *pale*, resulting in the modulation of the behavioral phase changes of locusts through post-transcriptional regulation (Figures S10). This miRNA-mediated mechanism of phenotypic plasticity induced by environmental fluctuations provides insight into the molecular basis of the phase changes in locusts.

The regulatory function of miR-133 in dopamine synthesis is shared among insects and vertebrates, but the corresponding mechanisms by which this regulation is achieved are different. In locusts, miR-133 regulates dopamine synthesis by directly targeting the 3′UTR of *pale*, and this regulation may be conserved in insects as the miR-133 target site is conserved. In contrast, miR-133 in vertebrates targets *Pitx3*, a transcription factor for tyrosine hydroxylase (encoded by *pale*), which regulates dopamine production [28]. However, no evidence of an association between miR-133 binding sites and insect *Ptx1* (a homolog to *Pitx3*) or the *Ptx1* regulation of *pale* (data not shown) has been reported. In addition to the dopamine pathway, other pathways are regulated by miR-133 in vertebrates, including heart regeneration and atrial remodeling [29], [30]. Given that miR-133 is conserved across a broad range of species, the function of this miRNA may also be conserved between insects and vertebrates. Therefore, miR-133 may also regulate multiple pathways in insects in addition to dopamine synthesis. Further studies are needed to verify this interpretation.

Fine-tuning the regulation of gene expression through acquired miRNA target sites in crucial genes contributes to species evolution [31]. Canonical miRNA target sites could be divided into two classes, namely the conserved and non-conserved target sites. In our study, the miR-133 target site in *henna* is not conserved across insect species, which implies that the miR-133 target site in *henna* may have been acquired after the divergence of the locust lineage from other insects. This miRNA target is correlated with the evolutionary emergence of the phase transition traits of locusts.

We found that miR-133 targets two genes, *henna* and *pale*, in the dopamine synthesis pathway of locusts. However, only the target site located in *pale* is conserved among insects, whereas the miR-133 target site in *henna* is unique to locusts. This observation suggests that the interactions between miR-133 and *henna* may have co-evolved with the phase transitions in locusts. miRNAs have been shown to exert their function through the coordinated inhibition of multiple targets within the same pathway [32]. The miRNA effect on any given target is usually modest, but its cumulative effect on multiple targets becomes phenotypically significant [33], [34]. Therefore, targeting multiple genes guarantees the miR-133-controlled phase transition, which may be necessary to regulate dopamine synthesis for a complete behavioral shift between the gregarious and solitary phases. Therefore, the simultaneous effects of a single miRNA on multiple targets in the same canonical pathway are advantageous.

In locusts, miR-133 targets *henna* in the coding region and *pale* in its 3′UTR. Additionally, miR-133 can only affect the protein, but not the mRNA expression level, of *pale*. The targeting of the *henna* coding region by miR-133 leads to mRNA degradation and reduces protein expression. Previous studies have indicated that miRNAs regulate gene expression by binding to the 3′UTR of target mRNAs, thereby resulting in mRNA degradation or translational repression [35]–[38]. In some cases, miRNAs can target sites in the 5′UTR and the CDS of mRNAs [39], [40]. Additional studies have attempted to elucidate the relative degree of contribution and the timing of translation inhibition and mRNA destabilization involved in miRNA-mediated silencing [41]–[43]. However, the molecular mechanisms underlying the determinant effects of translation inhibition and mRNA destabilization are far from being completely clarified.

In the present study, miR-133 was down-regulated as a result of increased population density, which indicates that this miRNA can be used as a sensor of population density. However, the mechanism of miR-133 regulation by population density remains unclear. A previous study [44] reported that p38 signaling is necessary for miR-133 transcription during early muscle regeneration. Moreover, the homolog of p38 has been identified from the locust transcriptome database [45], and qRT-PCR analysis showed that p38 expression is down-regulated in the brains of gregarious locusts (data not shown). Therefore, p38 may be a regulatory factor for miR-133 expression in response to changes in population density in locusts.

In summary, we report that miR-133 is a novel regulator promoting the migratory locust phase transitions by negatively regulating two predominant genes (*henna* and *pale*) in the dopamine pathway. miR-133 target sites are conserved among several insects and locusts, strongly suggesting that miR-133-mediated *pale* suppression is a component of a novel, biologically relevant, and evolutionarily conserved regulatory mechanism. This miRNA-mediated post-transcriptional regulation mechanism is particularly significant for understanding the behavioral aggregation of locusts and potentially provides new targets for controlling locust plagues worldwide.

## Materials and Methods

### Insects

Gregarious and solitary locusts were obtained from the same locust colonies, which were maintained at the Institute of Zoology, Chinese Academy of Sciences, China. Gregarious nymphs were reared in large, well-ventilated cages (40 cm×40 cm×40 cm) at a density of 500–1,000 insects per container for eight generations. Solitary nymphs were cultured alone in white metal boxes (10 cm×10 cm×25 cm) supplied with charcoal-filtered compressed air for more than eight generations before the experiment. Both colonies were reared under a 14∶10 light/dark photo regime at 30±2°C and were fed fresh wheat seedlings and bran [4].

### *In vitro* luciferase validation

The ∼400-bp sequences of the 3′ UTR and the CDS surrounding the predicted miR-133 target sites in *henna* and *pale*, respectively, were separately cloned into the psiCHECK-2 vector (Promega) using the XhoI and NotI sites. Mutagenesis PCR was performed at the miR-133 target sites. The miRNA expression plasmid contained the region encompassing the pre-miR-133 stem-loop inserted into pAc5.1/V5-HisA (Invitrogen). S2 cells in a 24-well plate were co-transfected with 800 ng of the luciferase reporter vector or the empty vector and 500 ng of the miR-133 expression plasmid using Lipofect (Tiangen). The activities of the firefly and *Renilla* luciferases were measured 48 h after transfection with the Dual-Glo Luciferase Assay System (Promega) using a luminometer (Promega).

### miRNA agomir and antagomir treatment *in vivo*

The miRNA agomir was a chemically modified, cholesterylated, stable miRNA mimic, and its *in vivo* delivery resulted in target silencing similar to the effects induced by the overexpression of endogenous miRNA [46]. Thus, ‘miRNA-133 overexpression’ in this study represents the expression activation of agomir-133. The miRNA antagomir was a chemically modified, cholesterol-conjugated, single-stranded RNA analog that complemented the miRNAs and could efficiently and specifically silence the endogenous miRNAs [47]. The brains of 2-day-old gregarious or solitary fourth instar nymphs were microinjected with agomir-133 or antagomir-133. A scrambled miRNA agomir/antagomir was used as a negative control. Briefly, fourth instar nymphs were placed in a Kopf stereotaxic frame specially adapted for locust surgery. A midline incision of approximately 2 mm was cut at the mid-point between the two antennae using Nevis scissors, and the underlying brain was exposed. Subsequently, 42 or 14 pmol of agomir-133 or antagomir-133 (200 µM; RiboBio) was injected into the brain. The agomir- or antagomir-negative controls (200 µM) were also injected into the gregarious or solitary locust brains (RiboBio). All injections were administered using a nanoliter injector (World Precision Instruments) with a glass micropipette tip under an anatomical lens. Treated nymphs were subjected to behavioral analysis 24 or 48 h later. Their brains were harvested, snap-frozen, and stored at −80°C.

### Protocerebrum isolation

Anatomical dissection was performed using a vibratome (Leica, VT1200S) to isolate the tissues from the protocerebrum, as indicated by an anatomic diagram of the locust brain. Briefly, the brain was dissected under an anatomical lens. The protocerebrum was placed on the slide at the boundary of the deutocerebrum and tritocerebrum and was cut using the vibratome. The levels of miR-133, *henna*, and *pale* in the isolated protocerebrums were analyzed using quantitative PCR (qPCR) or western blot analyses, and the interaction between miR-133 and *henna*/*pale* was analyzed using RNA immunoprecipitation analysis.

### Assays of quantitative PCR for mRNA and miRNA

Total RNA enriched for small RNAs was isolated using the mirVana miRNA isolation kit (Ambion). Moloney murine leukemia virus (M-MLV) reverse transcriptase (Promega) and a miRNA first-strand cDNA synthesis kit (Ambion) were used to prepare the Oligo(dT)-primed cDNA and stem-loop cDNA, respectively. The miRNAs and mRNAs were subjected to qPCR using the SYBR Green miRNA expression and gene expression assays, respectively, according to the manufacturer's instructions (Tiangen), on a LightCycler 480 instrument (Roche). Analysis of the PCR data was carried out using the 2^−ΔΔCt^ method of relative quantification. As endogenous controls, U6 snRNA and ribosomal protein *RP49* were used to quantify the miRNA and mRNA expression levels, respectively. Dissociation curves were determined for each miRNA and mRNA to confirm unique amplification. Table S3 shows the primers used for qPCR analysis.

### Western blot analysis

For protein analyses, affinity-purified polyclonal antibodies against *henna and pale* and a monoclonal antibody against Ago-1 were developed (Abmart), and the specificities of these antibodies were evaluated (Figures S11 and S12). The locust tissues were collected on a frozen cryostat and homogenized in lysis buffer (CoWin). The total protein content of the lysates was determined using a bicinchoninic acid protein assay kit (Thermo Scientific). The protein samples were separated by gel electrophoresis and then transferred to polyvinylidene difluoride (PVDF) membranes (Millipore). Non-specific binding sites on the membranes were blocked with 5% bovine serum albumin (BSA). The blots were incubated with the primary antibodies (rabbit anti-henna serum, 1∶500; rabbit anti-pale serum, 1∶700; mouse anti-Ago-1, 1∶1000) in PBS-T overnight at 4°C, washed, incubated with anti-rabbit IgG secondary antibody (1∶1000) (CoWin) for 1 h at room temperature, and then washed again. Immunological detection was subsequently carried out using a 5-bromo-4-chloro-3-indolyl phosphate/nitroblue tetrazolium substrate (Tiangen). The antibodies were stripped from the blots, which were then re-blocked and probed with an anti-β-actin antibody (CoWin). The intensities of the western blot signals were quantified using densitometry.

### Co-localization of miRNA and its targets by fluorescence *in situ* hybridization and immunohistochemical detection

A combined *in situ* analyses of miRNA-133 and *henna*/*pale* were detected in the brains of fourth instar nymphs by the colabeling of miRNA FISH and immunohistochemistry for miRNA target, according to the method described by Nuovo et al. [48]. An antisense locked nucleic acid (LNA) detection probe for miR-133 or a scrambled control (Exiqon) was labeled with double digoxigenin. The brains were fixed in 4% paraformaldehyde overnight. The paraffin-embedded brain tissue slides (5 µm thick) were deparaffinized in xylene, rehydrated with an ethanol gradient, digested with 20 µg/mL proteinase K (Roche) at 37°C for 10 min, and incubated with the LNA miR-133 probes or the scrambled control probe (2 pmol/µL) at 60°C for 5 min. The slides were then hybridized for 7–15 h at 37°C and were washed in 0.2× SSC and 2% BSA at 4°C for 5 min. The slides were incubated in anti-digoxigenin–alkaline phosphatase conjugate (1∶150 dilution) for 30 min at 37°C, followed by incubation with the HNPP substrate and TSA plus amplification (PerkinElmer) reagent working solution. After *in situ* hybridization, immunohistochemistry was performed to detect the miRNA targets. The slides were blocked with 5% BSA for 30 min and were incubated with anti-henna (1∶200) and anti-pale primary antibodies (1∶300) for 2 h. The slides were then washed, incubated for 1 h with Alexa Fluor-488 goat anti-rabbit secondary antibody (Life technologies), dehydrated, and cover slipped. miR-133 and its target signals were detected using a LSM 710 confocal fluorescence microscope (Zeiss).

### RNA immunoprecipitation (RIP) assays

The RIP assay was performed using a Magna RIP Quad kit (Millipore) with slight modifications. Briefly, the monoclonal antibody against Ago-1 protein was developed in mice (Abmart). Approximately 25 protocerebrums were collected and homogenized in ice-cold RIP lysis buffer. The homogenates were stored at −80°C overnight. Magnetic beads were pre-incubated with 5 µg of Ago-1 antibody (Abmart) or with 5 µg of normal mouse IgG (Millipore), which was used as a negative control. The frozen homogenates in the RIP lysate were thawed and centrifuged, and the supernatant was incubated with the magnetic bead–antibody complex at 4°C overnight. The immunoprecipitates were digested with protease K to remove the proteins and to release the RNAs. The RNAs were then purified and reverse-transcribed into cDNA using random hexamers. Using these cDNAs as templates, RT-PCR or qPCR was performed to quantify the *henna* and *pale* RNA. The supernatants of the RIP lysate (input) and the IgG controls were assayed to normalize the relative expression levels and ensure the specificity of the RNA–protein interactions.

### Behavioral assays

Behavioral assays were conducted in a rectangular arena (40 cm×30 cm×10 cm) with opaque walls, as described in a previous study [7]. The EthoVision system (Noldus) was used for video recording and behavioral data extraction [49]. The behavioral parameters were recorded and expressed as a mixture of behavioral or categorical markers [7]. To measure and quantify the behavioral phenotypes of the injected nymphs, a binary logistic regression model of the phase state was constructed using the SPSS 17.0 software for the gregarious and solitary fourth-instar locust nymphs [50]. A forward stepwise approach was performed to construct a binary logistic regression model: P_greg_ = e^n^/(1+e^n^), where *n* = β_0_+β_1_·X_1_+β_2_·X_2_+…+β_k_·X_k_, where P_greg_ indicates the probability of a locust being considered gregarious. P_greg_ = 1 indicates fully gregarious behavior, whereas P_greg_ = 0 indicates fully solitary behavior. This model shared similar features with a previous logistic model [50], [51]. The regression model of the migratory locust was used to discriminate the behavioral states of the locusts treated with the miRNA agomir or antagomir.

### Quantification of dopamine in the brain tissue extracts

The dopamine content of the locust brains was quantified using reverse-phase HPLC and ECD. The brain tissues of the locust nymphs were immediately dissected and stored in liquid nitrogen. Ten brains were homogenized and lysed in 400 µL of ice-cold 0.1 M perchloric acid for 10 min, after which the homogenates were centrifuged at 14,000 g for 10 min at 4°C. The supernatants were filtered through 0.45-µm filters. Approximately 40 µL of the supernatant was automatically loaded into the HPLC system, which contained a quaternary low-pressure pump system (Waters, e2695) with a C18 reversed-phase column (Atlantis dC18, 2.1 mm×150 mm, 3 µm). The electrochemical detector electrode potential was 800 mV. The mobile phase (pH, 3.00; temperature, 35°C; flow rate, 0.25 mL/min) was composed of 7% acetonitrile, 90 mM monobasic phosphate sodium, 50 mM citric acid, 2 mM octanesulfonic acid, 2 mM NaCl, and 50 µM EDTA. Data analysis was conducted using the Empower software (Waters Corporation). Dopamine was quantified by referring to an external standard.

### DNA constructs, cell transfections, and expression assays

Synthetic constructs were individually generated by cloning the sequence of HC1, HC2, PC1, or PC2 into the PAC-5.1/V5 His A vector (Invitrogen) at the EcoRI/Xbal sites. To generate HC1M and PC2M carrying the seed element mutations, four nucleotides in the seed region of the miRNA binding sites were mutated using the QuikChange II XL site-directed mutagenesis kit (Stratagene). S2 cells were co-transfected with the plasmid expression vectors (HC1, HC2, HC1M, PC1, PC2, PC2M, or empty vector) and agomir-133 or agomir-NC at a 1∶4 ratio using the Lipofectamine 2000 reagent (Invitrogen) according to the manufacturer's instructions.

The mRNA levels of *henna* and *pale* in the co-transfected S2 cells were investigated after 48 h of transfection. Total RNA was extracted using an RNeasy Mini Kit (Qiagen) according to the manufacturer's protocol. cDNA was reverse-transcribed from 2 µg of total RNA using M-MLV reverse transcriptase (Promega). *β-Actin* was used as an internal control. qPCR was performed using a SYBR Green amplification kit (Tiangen) according to the manufacturer's instructions.

To determine the protein levels of *henna* and *pale* in the co-transfected S2 cells, the cells were harvested and lysed in lysis buffer (Tiangen). Approximately 20 µg of total protein was subjected to 10% sodium dodecyl sulfate-polyacrylamide gel electrophoresis and then transferred to PVDF membranes, as previously described.

### Behavioral rescue experiments *in vivo*

The rescue experiments were performed according to the method of miRNA agomir and antagomir treatment *in vivo*. In gregarious locusts, the brains of 2-day-old fourth instar nymphs were microinjected with 42 pmol of miR-133 agomir (200 µM, RiboBio). Twenty-four hours later, the gregarious nymphs were co-injected with 20 µg of *R*-(-)-apomorphine or a saline control. Then, 24 h after *R*-(-)-apomorphine injection, the nymphs were subjected to behavioral analysis. The brains of the solitary locusts were microinjected with 42 pmol of miR-133 antagomir (200 µM; RiboBio). Twenty-four hours later, the solitary nymphs were co-injected with 5 µg of dsHenna/dsPale or a dsGFP control. Then, 24 h after dsRNA injection, the nymphs were subjected to behavioral analysis.

### Statistical analysis

The SPSS 17.0 software (SPSS Inc.) was used for statistical analysis. The differences between the treatments were compared using either Student's *t*-test or one-way analysis of variance (ANOVA) followed by Tukey's test for multiple comparisons. The Mann–Whitney *U* test was used to analyze the behavioral data due to its non-normal distribution characteristics. *p*<0.05 was considered statistically significant. All results are expressed as the mean ± SEM.

## Supporting Information

Figure S1 Targeting sites of miR-133 in *henna* and *pale*. In locusts, miR-133 was bound to *henna* at a partial complementary site in its coding region, and it was bound to *pale* at a partial complementary site in its 3′ untranslated region.
(TIF)

Figure S2 miR-994 expression in the brains of gregarious (G) and solitary (S) locusts as determined by qRT-PCR. The data are presented as the mean ± SEM (*n* = 6).
(TIF)

Figure S3 miR-133 and *henna*/*pale* co-localize in the protocerebrum. (A) A diagram of the locust brain. MB: mushroom body; PB: protocerebral bridge; CB: central body; AL: antennal lobe. (B) An anatomic diagram of the locust brain. The brain consists of the protocerebrum (PR), deutocerebrum (DE), and tritocerebrum (TR). (C, D) The combined *in situ* analyses of miRNA-133 and *henna*/*pale* by the co-labeling of miRNA FISH and immunohistochemistry for the miRNA target to determine the co-localization of miR-133 and *henna*/*pale* in the locust brain. The arrows specifically indicate the areas where miR-133 (red) and *henna*/*pale* (green) were co-localized in the locust brain. The images were visualized using an LSM 710 confocal fluorescence microscope at ×20 magnification (Zeiss).
(TIF)

Figure S4 miR-133 expression is negatively correlated with the expression of *henna* and *pale* in the protocerebrum. (A) The expression levels of miR-133 in the protocerebrum of gregarious (GP) and solitary locusts (SP) were determined using qPCR. (B, C) The expression levels of *henna* and *pale* in the protocerebrum of gregarious (GP) and solitary locusts (SP) were determined using qPCR (B) and western blot analyses (C). The qPCR data are presented as the mean ± SEM (*n* = 6). The western blot bands were quantified using densitometry and are expressed as the mean ± SEM (*n* = 4). **p<0.01; ***p<0.005.
(TIF)

Figure S5 miR-133 expression in the brains every day (D1–5: Days 1–5) during the fourth instar nymph stage as determined by qRT-PCR. All data are presented as the mean ± SEM (*n* = 6) of one representative experiment.
(TIF)

Figure S6 Effect of miR-133 overexpression or silencing on miR-133 expression. (A) miR-133 expression was quantified using qRT-PCR 24 h after the gregarious locust brains were treated with 42 pmol of agomir-133. (B) miR-133 expression was quantified using qRT-PCR 24 h after the solitary locust brains were treated with 42 pmol of antagomir-133. The data are shown as the mean ± SEM (*n* = 6) of one representative experiment. ***p<0.005.
(TIF)

Figure S7 Effect of miR-133 overexpression or silencing on the expression of other miRNAs. The expression levels of other miRNAs (miR-7 and miR-252) were quantified using qPCR 48 h after the gregarious (G) and solitary (S) locust brains were treated with 42 pmol of agomir and antagomir-133, respectively. All data are shown as the mean ± SEM (*n* = 6) of one representative experiment.
(TIF)

Figure S8 Effect of miR-133 overexpression or silencing on the expression of *henna*. (A) *Henna* expression was quantified using qRT-PCR 24 or 48 h after treatment of gregarious locust brains with 14 or 42 pmol agomir-133. (B) *Henna* expression was quantified using qRT-PCR 24 or 48 h after treatment of solitary locust brains with 14 or 42 pmol antagomir-133. The data are shown as the mean ± SEM (*n* = 6) of one representative experiment. *p<0.05; **p<0.01.
(TIF)

Figure S9 miR-133 fosters the phase transition phenotype of the migratory locust. (A) The effects of 42 pmol of agomir-133 on the behavior of the gregarious locusts were studied 24 h after injection. (B) The effects of 42 pmol of antagomir-133 on the behavior of the solitary locusts were studied 24 after injection. P_greg_, probabilistic metric of gregariousness. The vertical lines indicate the median P_greg_ values.
(TIF)

Figure S10 Model of the miR-133-mediated dopamine pathway associated with the phase changes of the migratory locust. miR-133 controls dopamine production by regulating *henna* and *pale* in the locust brain.
(TIF)

Figure S11 Validation of the monoclonal antibody against Ago-1 protein. Western blot analysis of Ago-1 was performed in tissue lysates (input) and Ago-1 immunoprecipitates (IP). Mouse IgG was used as a negative control.
(TIF)

Figure S12 Validation of the polyclonal antibodies against the henna and pale proteins. RNAi-induced knockdown of *henna* (A) and *pale* (B) was used to validate the antibody specificity. RNAi GFP was used a control. The arrows indicate the single and specific bands of the expected size.
(TIF)

Table S1 miRNAs searched in *Drosophila* using the TargetScan database.
(XLS)

Table S2 miRNAs searched in *Locusta migratoria* as predicted by the miRanda software.
(XLS)

Table S3 Primers used for the qPCR analysis of *pale*, *henna*, *RP49*, *miR-133*, miR-994, *miR-7*, *miR-252*, and *U6* and in the construction of the HC1, HC2, HC1M, PC1, PC2, and PC2M.
(XLS)
