# Supplementary figures and images for: MicroRNA-133 Inhibits Behavioral Aggregation by Controlling Dopamine Synthesis in Locusts
Source: PLoS Genet. 2014 Feb 27;10(2):e1004206. doi: 10.1371/journal.pgen.1004206 (PMC3937255; doi:10.1371/journal.pgen.1004206)

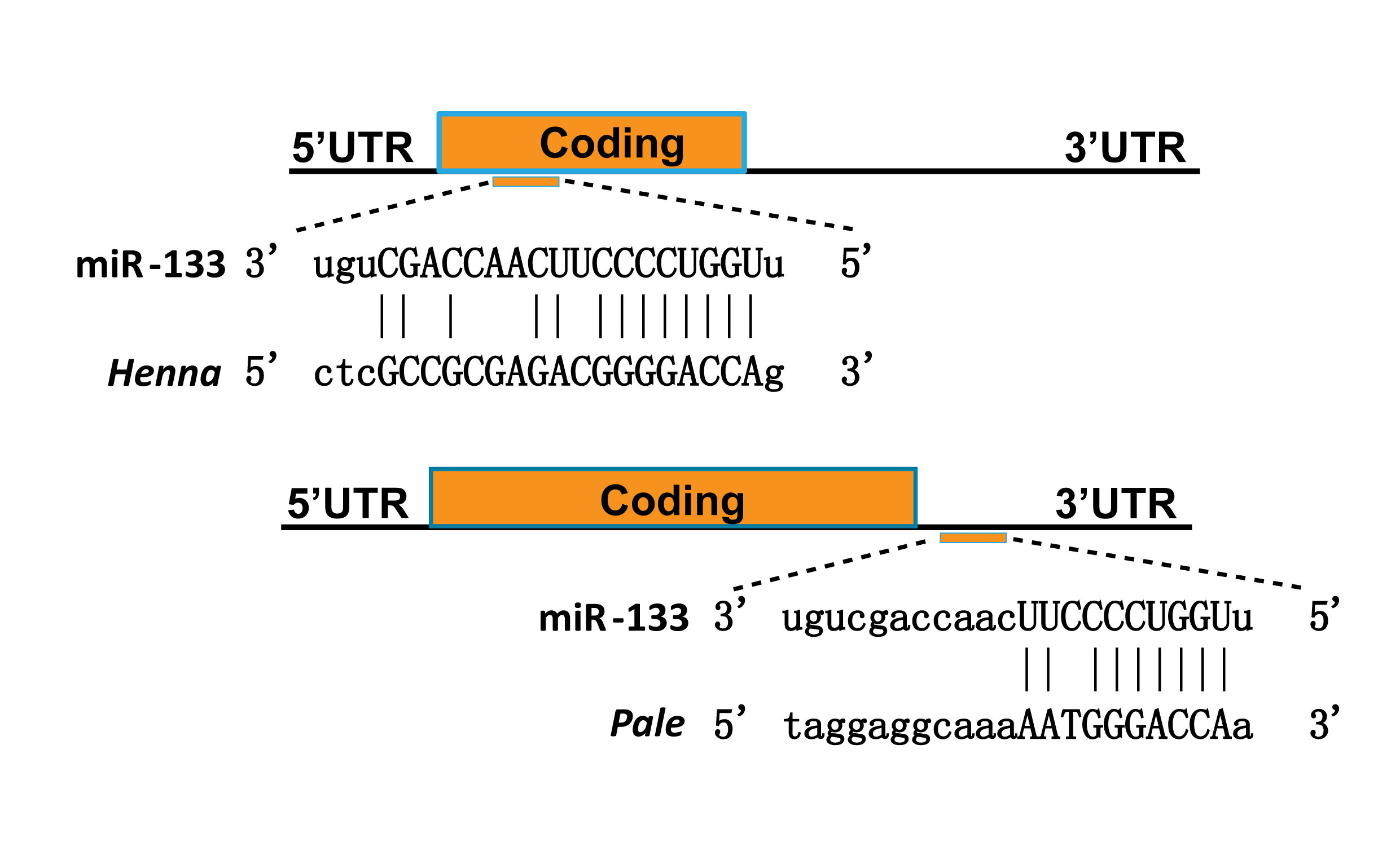

Supplement: Figure S1 — Targeting sites of miR-133 in henna and pale. In locusts, miR-133 was bound to henna at a partial complementary site in its coding region, and it was bound to pale at a partial complementary site in its 3′ untranslated region. (TIF) [file pgen.1004206.s001.tif]

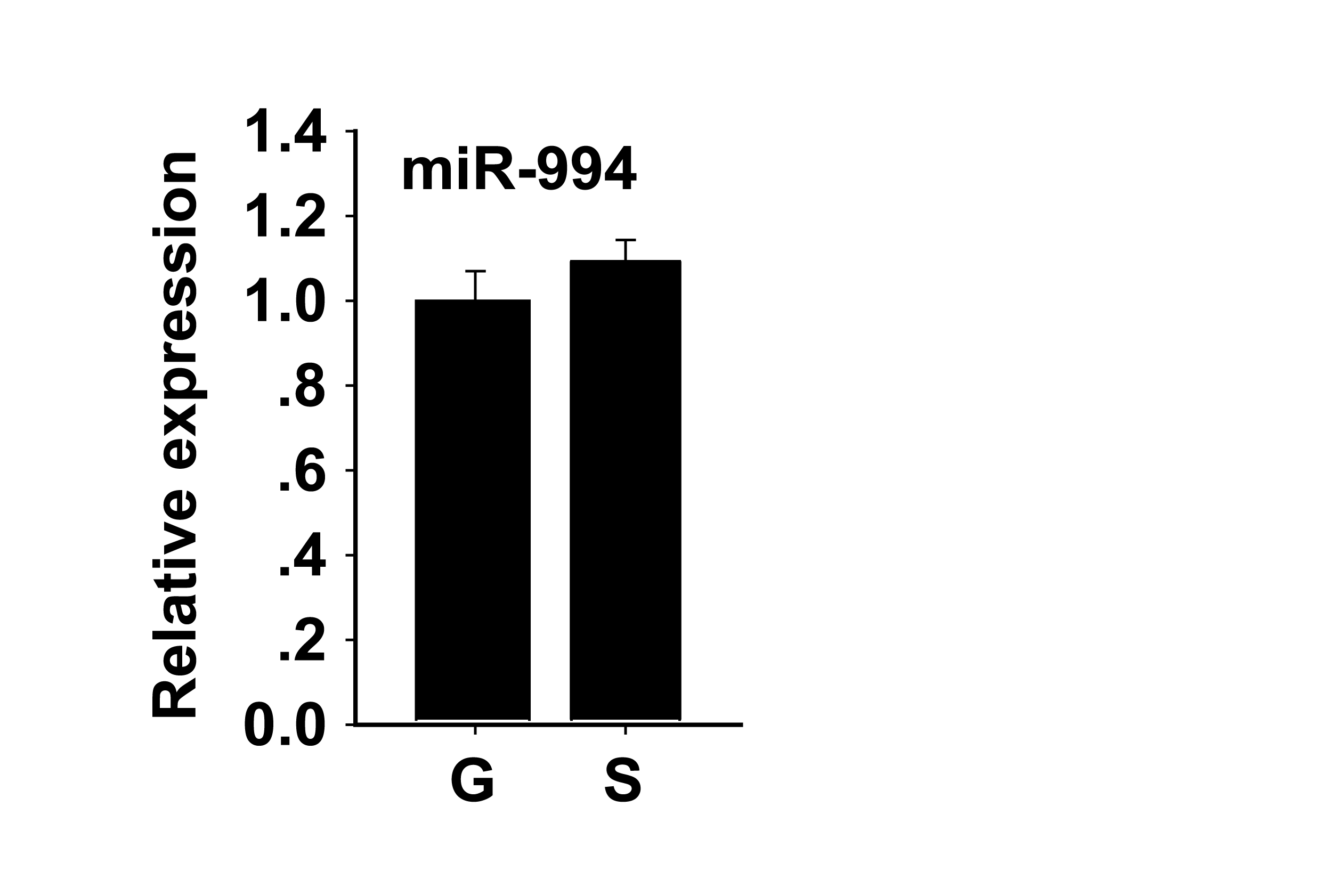

Supplement: Figure S2 — miR-994 expression in the brains of gregarious (G) and solitary (S) locusts as determined by qRT-PCR. The data are presented as the mean ± SEM (n = 6). (TIF) [file pgen.1004206.s002.tif]

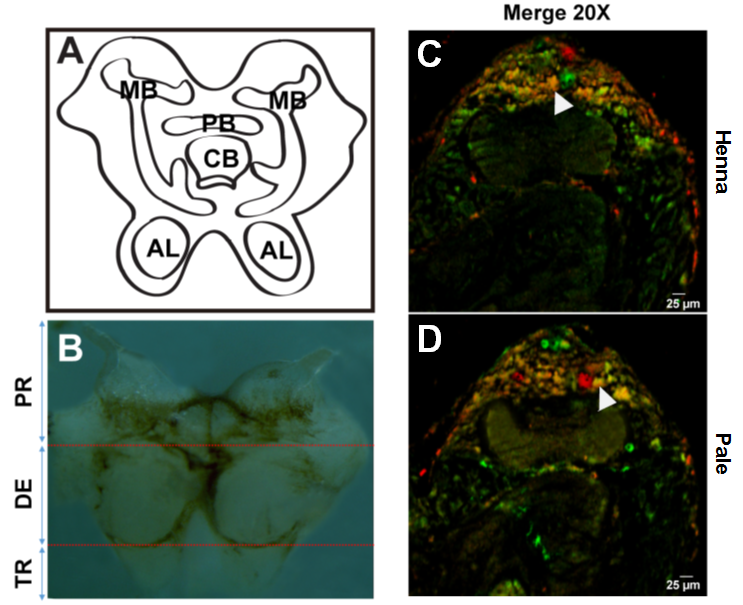

Supplement: Figure S3 — miR-133 and henna/pale co-localize in the protocerebrum. (A) A diagram of the locust brain. MB: mushroom body; PB: protocerebral bridge; CB: central body; AL: antennal lobe. (B) An anatomic diagram of the locust brain. The brain consists of the protocerebrum (PR), deutocerebrum (DE), and tritocerebrum (TR). (C, D) The combined in situ analyses of miRNA-133 and henna/pale by the co-labeling of miRNA FISH and immunohistochemistry for the miRNA target to determine the co-localization of miR-133 and henna/pale in the locust brain. The arrows specifically indicate the areas where miR-133 (red) and henna/pale (green) were co-localized in the locust brain. The images were visualized using an LSM 710 confocal fluorescence microscope at ×20 magnification (Zeiss). (TIF) [file pgen.1004206.s003.tif]

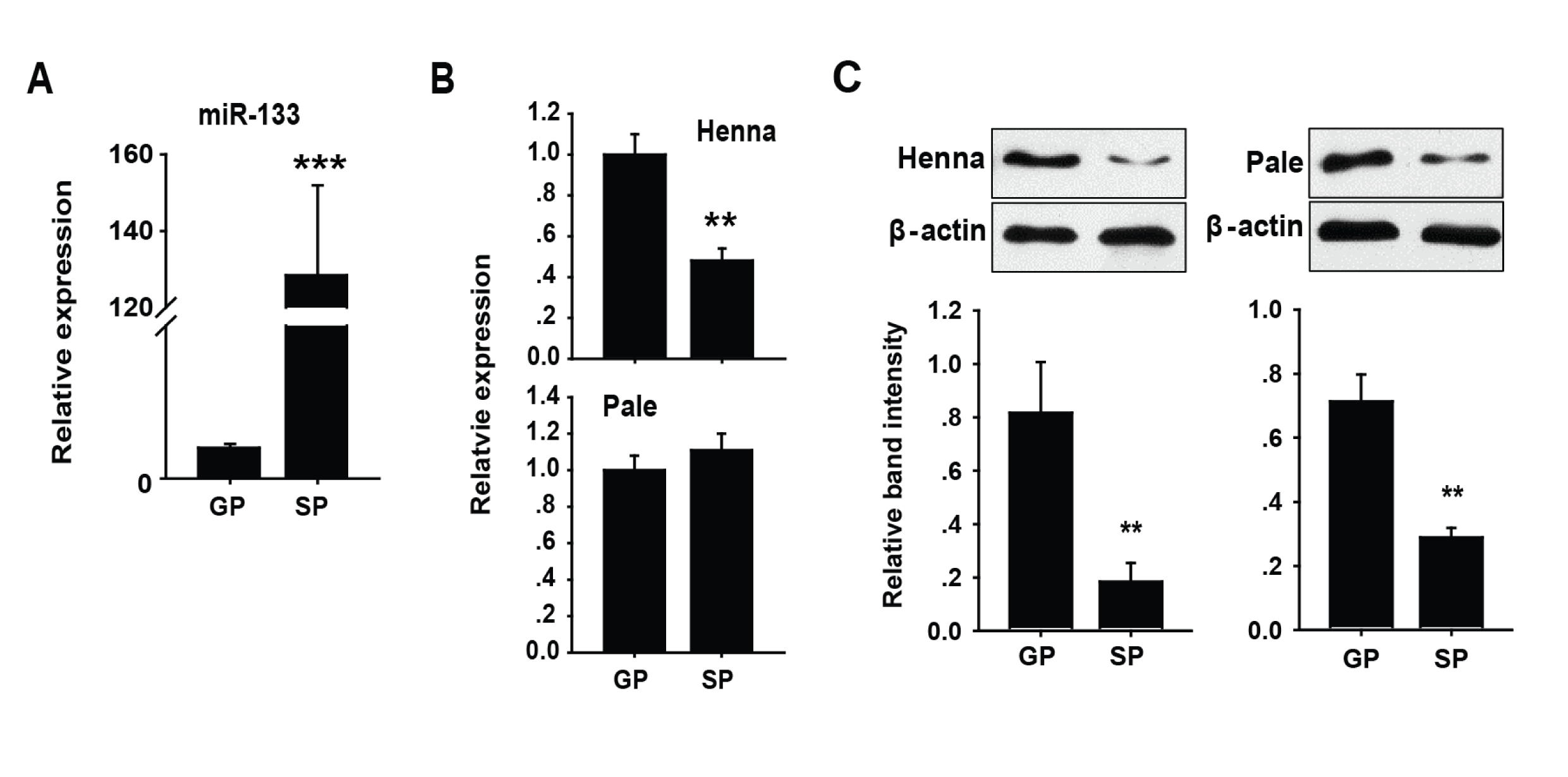

Supplement: Figure S4 — miR-133 expression is negatively correlated with the expression of henna and pale in the protocerebrum. (A) The expression levels of miR-133 in the protocerebrum of gregarious (GP) and solitary locusts (SP) were determined using qPCR. (B, C) The expression levels of henna and pale in the protocerebrum of gregarious (GP) and solitary locusts (SP) were determined using qPCR (B) and western blot analyses (C). The qPCR data are presented as the mean ± SEM (n = 6). The western blot bands were quantified using densitometry and are expressed as the mean ± SEM (n = 4). **p<0.01; ***p<0.005. (TIF) [file pgen.1004206.s004.tif]

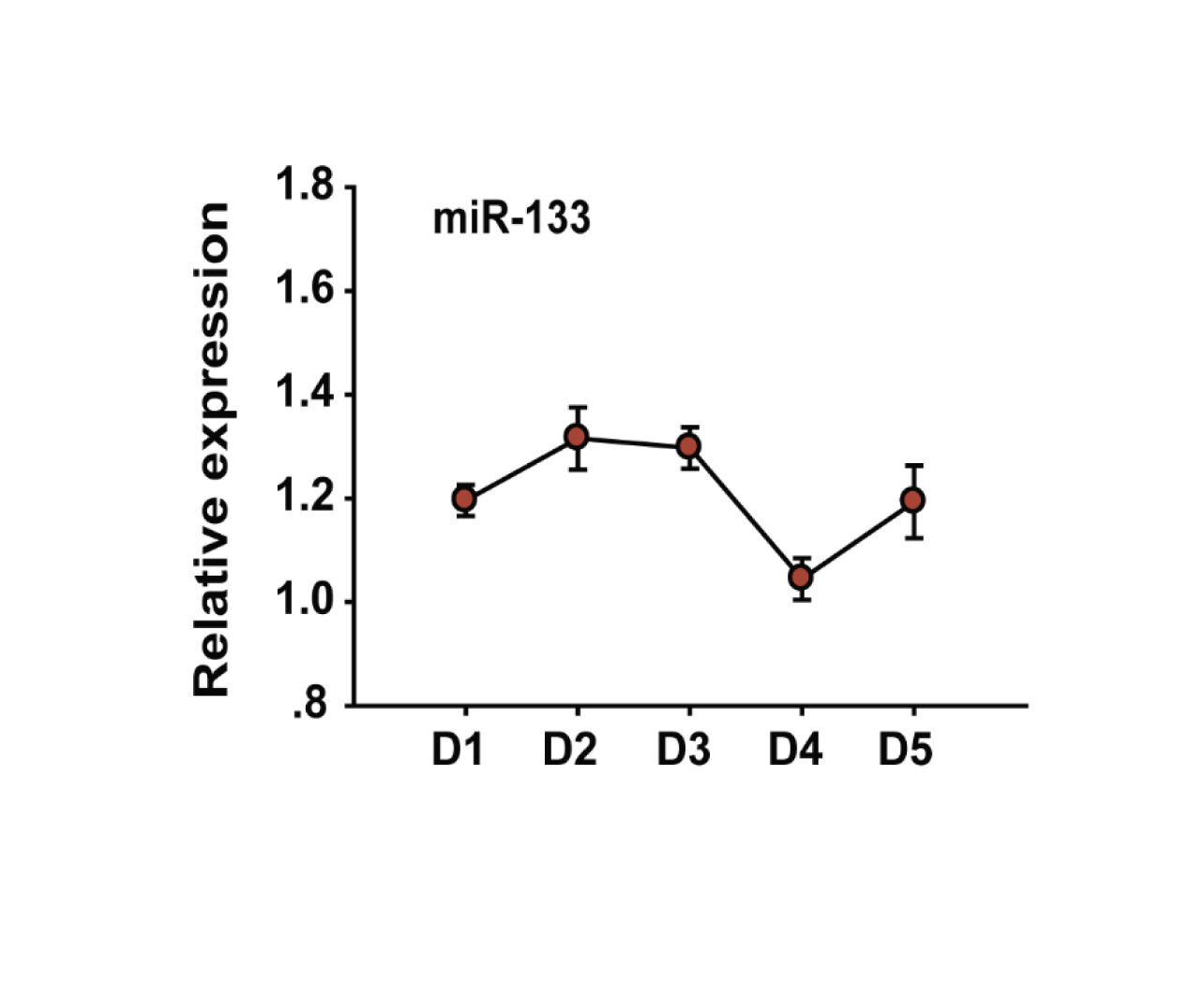

Supplement: Figure S5 — miR-133 expression in the brains every day (D1–5: Days 1–5) during the fourth instar nymph stage as determined by qRT-PCR. All data are presented as the mean ± SEM (n = 6) of one representative experiment. (TIF) [file pgen.1004206.s005.tif]

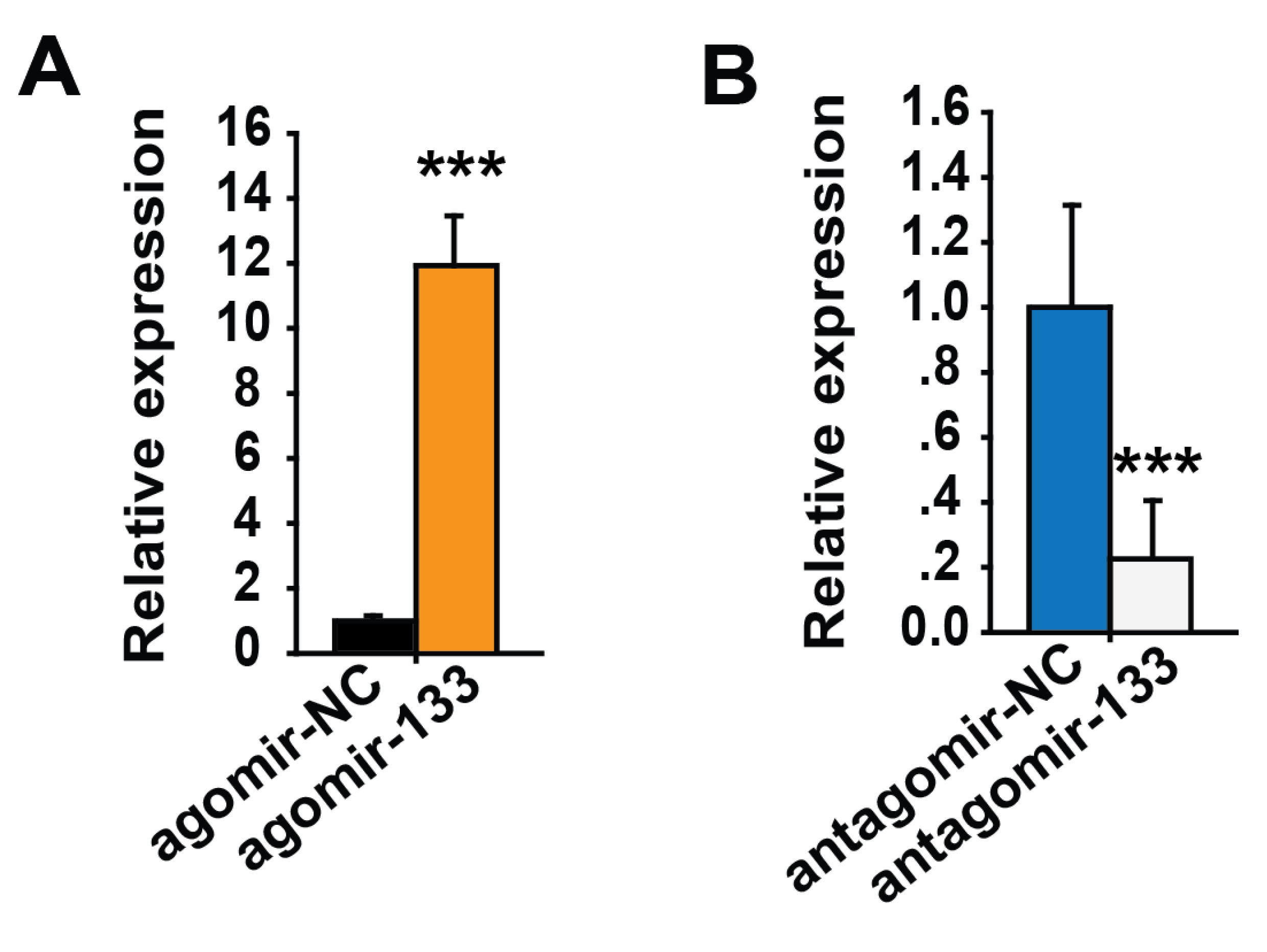

Supplement: Figure S6 — Effect of miR-133 overexpression or silencing on miR-133 expression. (A) miR-133 expression was quantified using qRT-PCR 24 h after the gregarious locust brains were treated with 42 pmol of agomir-133. (B) miR-133 expression was quantified using qRT-PCR 24 h after the solitary locust brains were treated with 42 pmol of antagomir-133. The data are shown as the mean ± SEM (n = 6) of one representative experiment. ***p<0.005. (TIF) [file pgen.1004206.s006.tif]

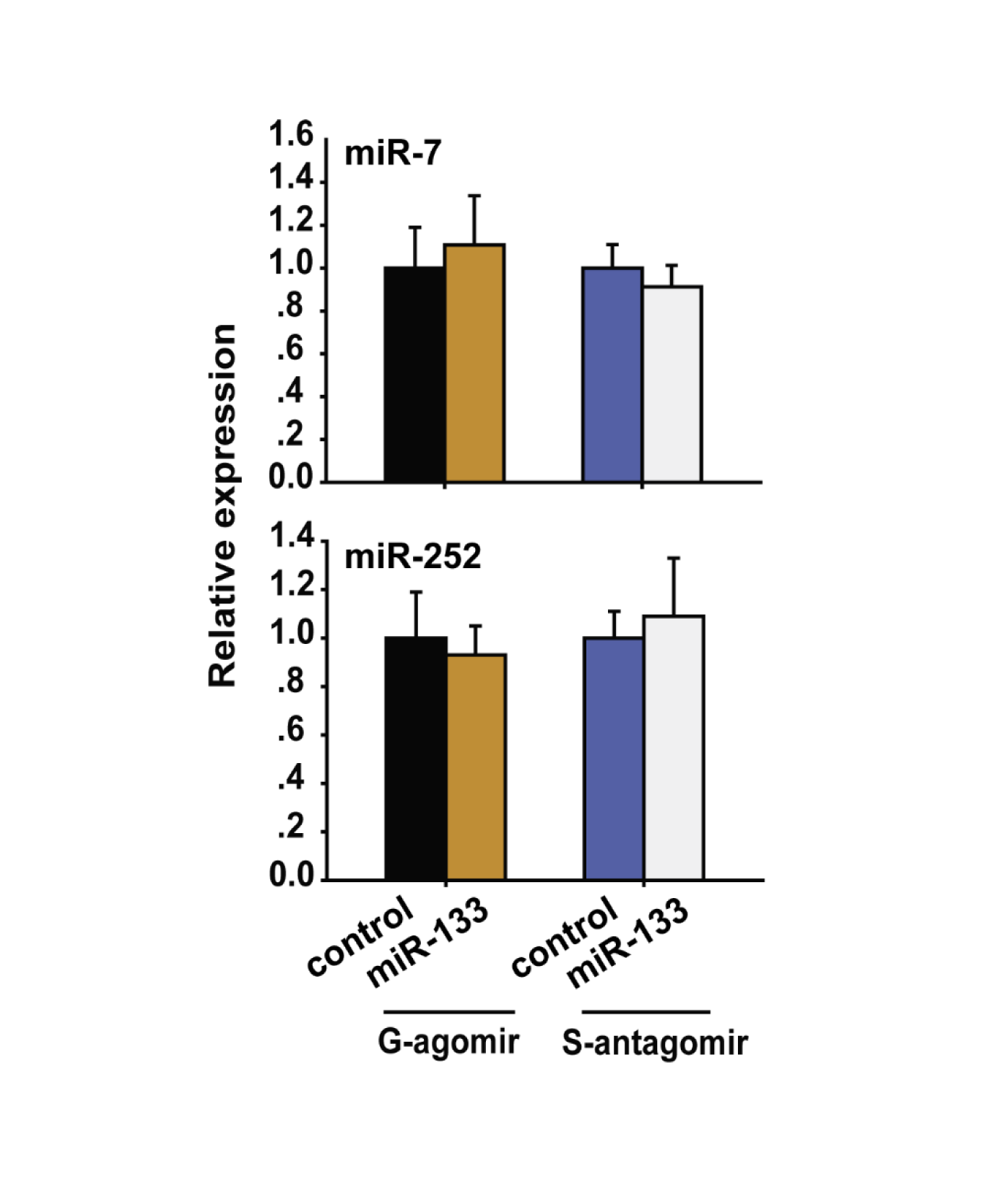

Supplement: Figure S7 — Effect of miR-133 overexpression or silencing on the expression of other miRNAs. The expression levels of other miRNAs (miR-7 and miR-252) were quantified using qPCR 48 h after the gregarious (G) and solitary (S) locust brains were treated with 42 pmol of agomir and antagomir-133, respectively. All data are shown as the mean ± SEM (n = 6) of one representative experiment. (TIF) [file pgen.1004206.s007.tif]

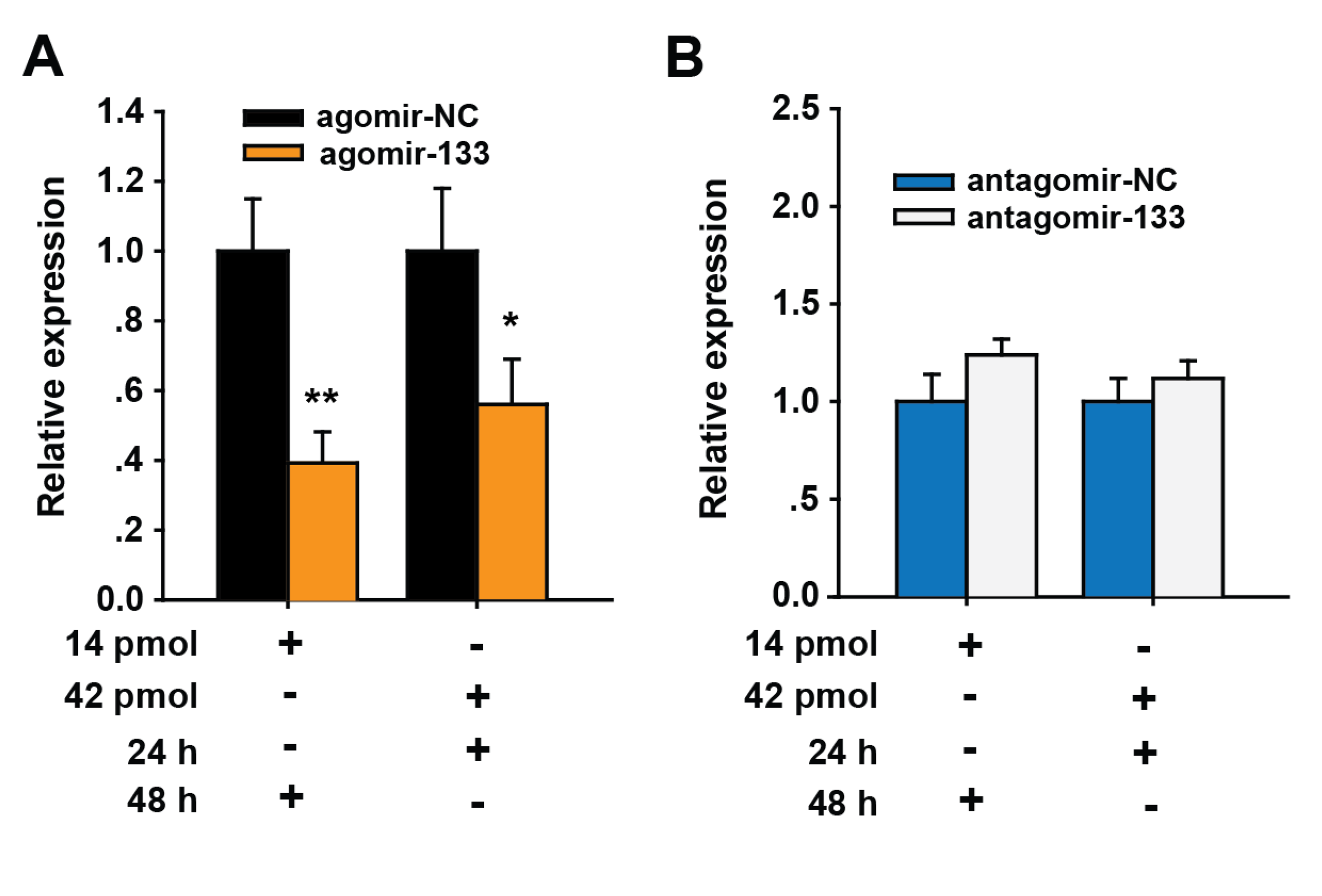

Supplement: Figure S8 — Effect of miR-133 overexpression or silencing on the expression of henna. (A) Henna expression was quantified using qRT-PCR 24 or 48 h after treatment of gregarious locust brains with 14 or 42 pmol agomir-133. (B) Henna expression was quantified using qRT-PCR 24 or 48 h after treatment of solitary locust brains with 14 or 42 pmol antagomir-133. The data are shown as the mean ± SEM (n = 6) of one representative experiment. *p<0.05; **p<0.01. (TIF) [file pgen.1004206.s008.tif]

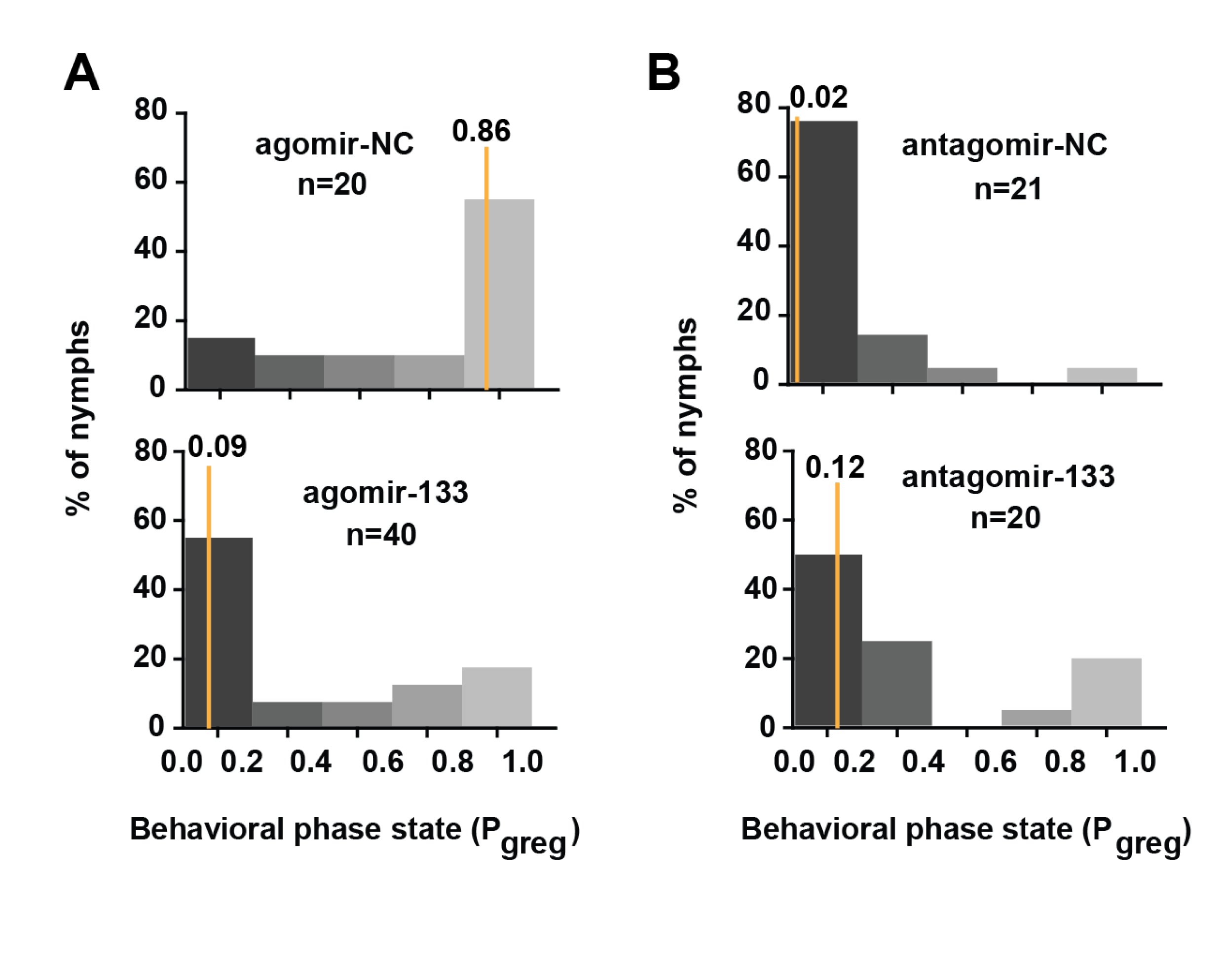

Supplement: Figure S9 — miR-133 fosters the phase transition phenotype of the migratory locust. (A) The effects of 42 pmol of agomir-133 on the behavior of the gregarious locusts were studied 24 h after injection. (B) The effects of 42 pmol of antagomir-133 on the behavior of the solitary locusts were studied 24 after injection. Pgreg, probabilistic metric of gregariousness. The vertical lines indicate the median Pgreg values. (TIF) [file pgen.1004206.s009.tif]

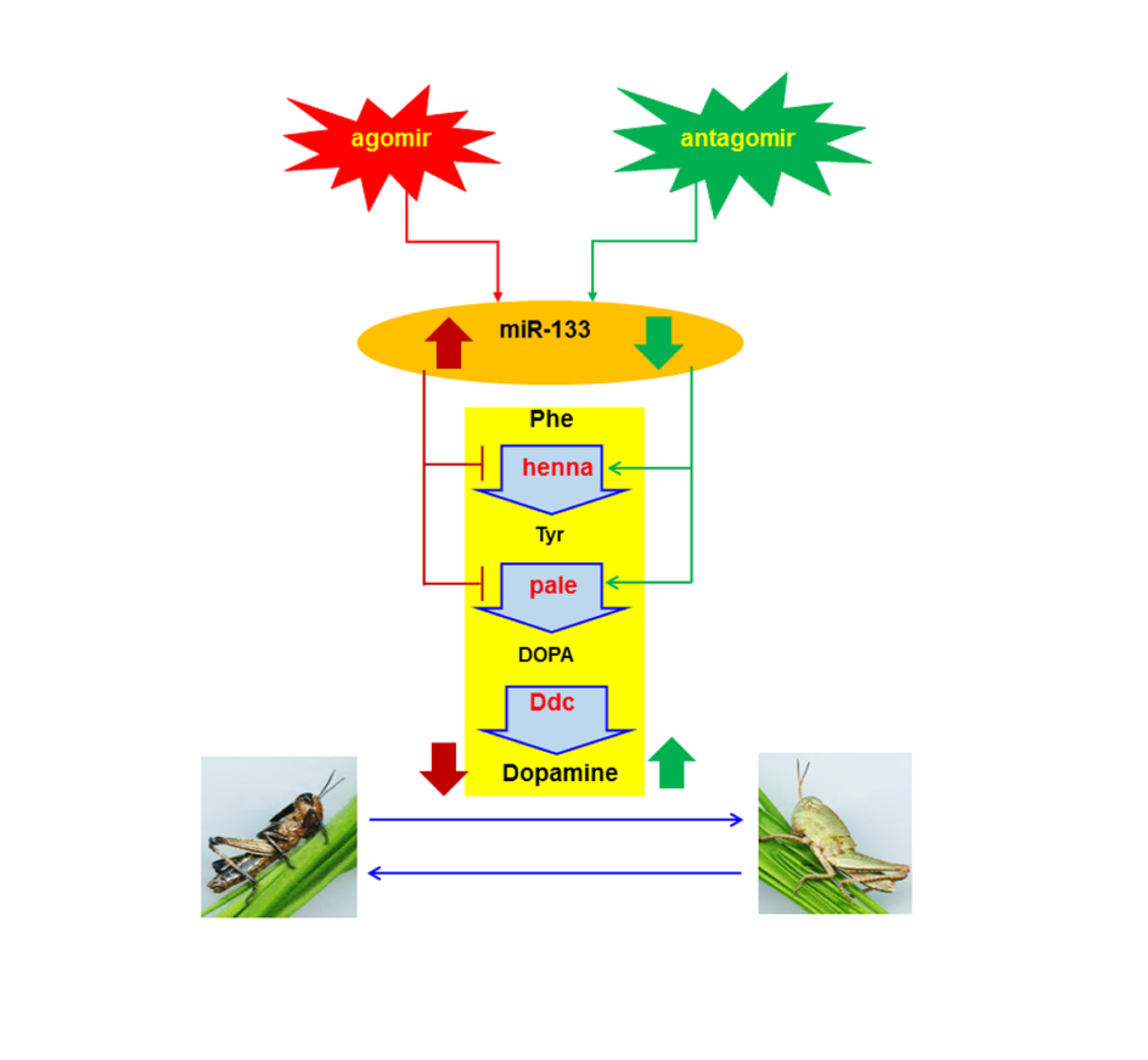

Supplement: Figure S10 — Model of the miR-133-mediated dopamine pathway associated with the phase changes of the migratory locust. miR-133 controls dopamine production by regulating henna and pale in the locust brain. (TIF) [file pgen.1004206.s010.tif]

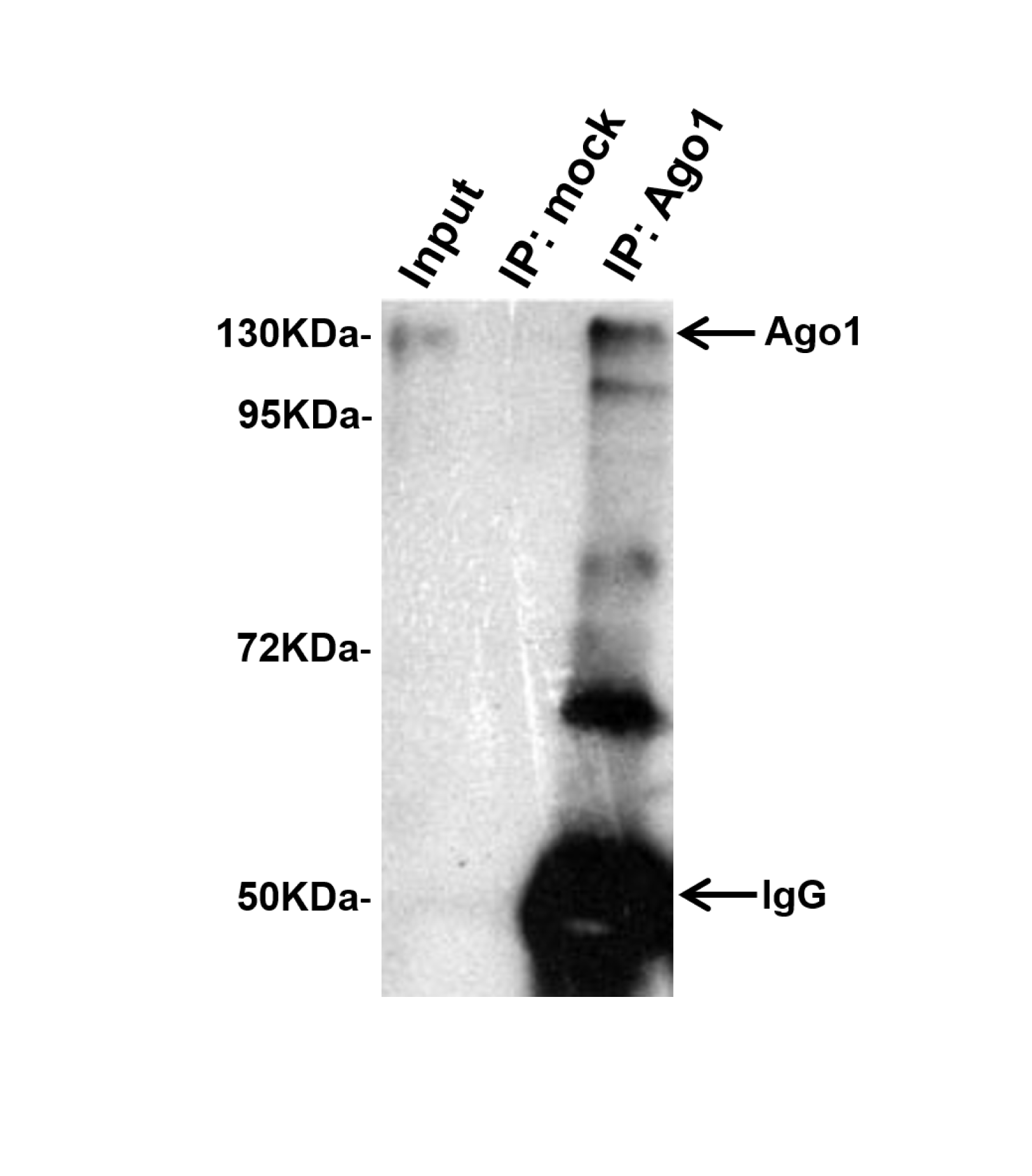

Supplement: Figure S11 — Validation of the monoclonal antibody against Ago-1 protein. Western blot analysis of Ago-1 was performed in tissue lysates (input) and Ago-1 immunoprecipitates (IP). Mouse IgG was used as a negative control. (TIF) [file pgen.1004206.s011.tif]

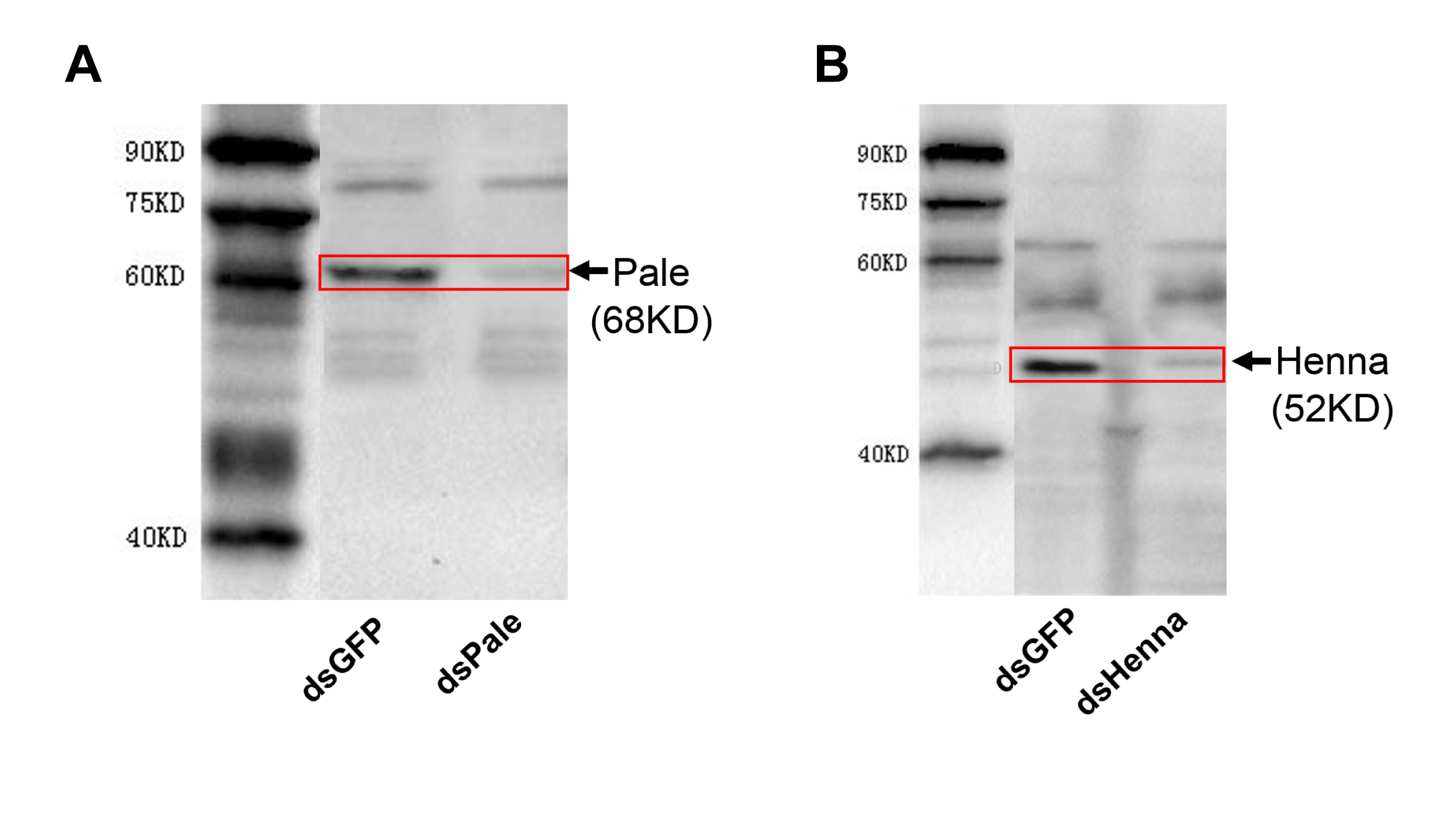

Supplement: Figure S12 — Validation of the polyclonal antibodies against the henna and pale proteins. RNAi-induced knockdown of henna (A) and pale (B) was used to validate the antibody specificity. RNAi GFP was used a control. The arrows indicate the single and specific bands of the expected size. (TIF) [file pgen.1004206.s012.tif]
